# Phase Separation of SF3B1 Serves as a Critical Post‐Transcriptional Regulator During Early Mouse Embryogenesis

**DOI:** 10.1002/advs.77605

**Published:** 2026-09-08

**Authors:** Kang Zhao, Ting‐Yu Han, Yan‐Li Cheng, Yi‐Dan Zhang, Yu‐Wei Zhang, JinJian Guo, ShaoJun Zhang, WenZe Huang, Jing Zhang, Pei‐Yu Liao, Ying Xin, ChuanChen Chu, Qing‐Yuan Sun, ZhiZhen Liu, Xiang‐Hong Ou, Jun Xie

**Affiliations:** ^1^ Department of Biochemistry and Molecular Biology Shanxi Key Laboratory of Birth Defect and Cell Regeneration Shanxi Medical University Taiyuan China; ^2^ Key Laboratory of Coal Environmental Pathogenicity and Prevention (Shanxi Medical University) Ministry of Education Taiyuan China; ^3^ School of Life Science Shanxi Normal University Taiyuan China; ^4^ Guangzhou Key Laboratory of Metabolic Diseases and Reproductive Health Guangdong‐Hong Kong Metabolism & Reproduction Joint Laboratory Reproductive Medicine Center Guangdong Second Provincial General Hospital of Jinan University Guangzhou China; ^5^ Beijing Advanced Innovation Center for Structural Biology & Frontier Research Center for Biological Structure School of Life Sciences Tsinghua University Beijing China; ^6^ Tsinghua‐Peking Center for Life Sciences Beijing China; ^7^ The Second School of Clinical Medicine Southern Medical University Guangzhou China

**Keywords:** alternative splicing, biology, blastocyst, cell biology, embryonic stem cell, regulator, rna splicing, spliceosome, splicing factor, transcription factor

## Abstract

During early mammalian embryogenesis, totipotent zygotes and early blastomeres undergo extensive post‐transcriptional regulation during the establishment of the first cell lineages; however, the functional contribution of alternative splicing to embryonic compaction and blastulation remains poorly understood. Here, we show that SF3B1, a core component of the spliceosome, is upregulated from the 4‐cell stage and mediates highly dynamic splicing programs. Depletion of SF3B1 results in developmental arrest at the morula stage, accompanied by widespread transcriptomic dysregulation characterized by aberrant expression of transcription factors that impede pluripotency transition. Alternative splicing analysis further identifies that aberrantly spliced transcripts were significantly enriched in genes involved in cell cycle regulation, such as *Cdk11b* and *Ccnb1*. Importantly, we demonstrate that SF3B1 undergoes intrinsic, IDR‐driven liquid‐liquid phase separation both in vitro and in vivo, forming nuclear condensates in oocytes and early embryos, which is essential for successful development to the blastocyst stage. Together, our findings reveal that phase separation mediated SF3B1 splicing activity is a critical regulator of early mouse embryonic development.

## Graphical abstract

Phase separation of SF3B1 acts as a key regulatory mechanism for dynamic alternative splicing throughout early mouse embryogenesis. Its absence triggers extensive splicing errors, which induce persistent DNA damage, defective cell cycle progression, and failed cell lineage commitment, and ultimately hinder the normal growth and development of preimplantation blastocysts in mammalian embryos.

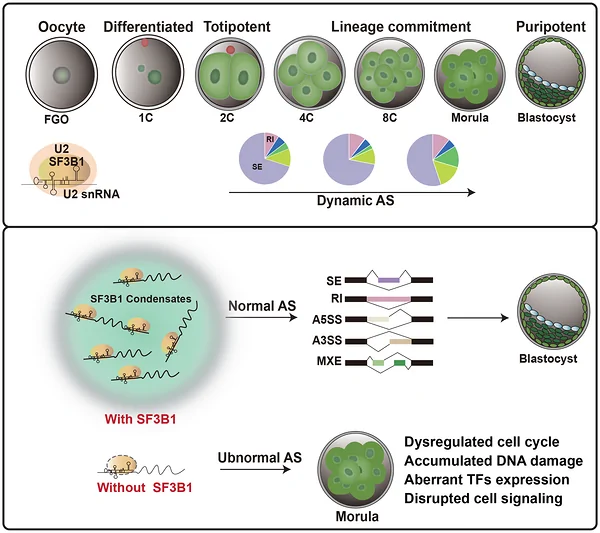

## 1 Introduction

Mammalian development begins with the zygote, which undergoes successive rounds of cleavage, followed by blastomere compaction to form a blastocyst [1]. Despite profound morphological and molecular remodeling during cleavage, blastomeres up to the 8‐cell stage maintain the capacity to contribute to both embryonic and extraembryonic lineages and are thus broadly considered totipotent [2, 3, 4]. Initially totipotent blastomeres undergo the first cell‐fate decision, giving rise to the pluripotent inner cell mass and the trophectoderm, which respectively contribute to the embryo proper and extraembryonic tissues [5]. Embryos undergo extensive transcriptomic reprogramming and are tightly coordinated by asymmetric division, biochemical signaling pathways, epigenetic modifications, and TF networks [6]. However, how post‐transcriptional regulation mechanistically underlies the first lineage segregation remains unclear.

Pre‐messenger RNA (pre‐mRNA) splicing generates mature mRNAs through the removal of introns and the ligation of exons, catalyzed by the spliceosome, a highly dynamic ribonucleoprotein complex that mediates intron excision and exon ligation through recognition of conserved 5′ and 3′ splice sites, producing diverse RNA and protein isoforms [7]. Nearly 95% of multi‐exon human genes undergo alternative splicing (AS), markedly expanding proteomic complexity [8]. In particular, RNA splicing has emerged as a critical determinant of oocyte competence and embryonic genome activation [9, 10, 11, 12]. Aberrant RNA splicing has been shown to reduce oocyte quality and impair the developmental potential of embryos derived from aged mouse oocytes [13, 14]. Notably, pre‐mRNA splicing is tightly coupled to transcription, with RNA polymerase II dynamics, nucleosome positioning, chromatin architecture, and epigenetic modifications jointly contributing to shaping splicing decisions [15]. Dynamic transcriptional and epigenetic changes occur in early embryos prior to the first cell fate decision [16, 17]. Several alternative splicing factors have been identified as regulators of pluripotent gene activity [18, 19]. However, the dynamic splicing profiles of this critical stage and how spliceosomal regulation modulates embryonic developmental potential remain to be clarified.

The SF3B protein family, consisting of key components including SF3B1, SF3B3, SF3B5, SF3B6, and PHF5A [20], comprises the core subcomplex of U2 small nuclear ribonucleoprotein (U2 snRNP), a critical part of the spliceosome that catalyzes pre‐mRNA splicing. SF3B1 is the largest subunit by molecular weight and contains extended intrinsically disordered regions (IDRs), a feature frequently associated with liquid‐liquid phase separation (LLPS), which may underlie its ability to form biomolecular condensates. In general, biomolecular condensates arise via weak multivalent interactions between proteins and nucleic acids [21], and participate in the spatiotemporal regulation of nuclear gene expression, encompassing key processes including transcription and splicing [22]. During co‐transcriptional splicing, enhancers and promoters facilitate co‐condensate formation by enriching transcription factors, while nascent RNAs locally recruit various RBPs to form RNA‐processing condensates, such as nuclear speckles [23, 24]. The capacity of RBPs to undergo LLPS serves as a core mechanism for globally modulating splicing outcomes and enhancing gene regulatory potential [25, 26, 27]. However, how spliceosomal components are spatially and temporally regulated within early embryos remains unclear.

In this study, we show that dynamic alternative splicing events occur during the first cell‐fate decision. We demonstrate that depletion of SF3B1 causes developmental arrest at the morula stage by disrupting a transcription factor (TF) network that normally drives pluripotency exit, and its splice‐switching activity controls the cell cycle. Furthermore, we establish that the phase‐separated SF3B1 condensates are necessary for early mouse embryonic development.

## 2 Results

### 2.1 Dynamic Alternative Splicing Accompanies Mouse Pluripotency Transitions

**FIGURE 1:**
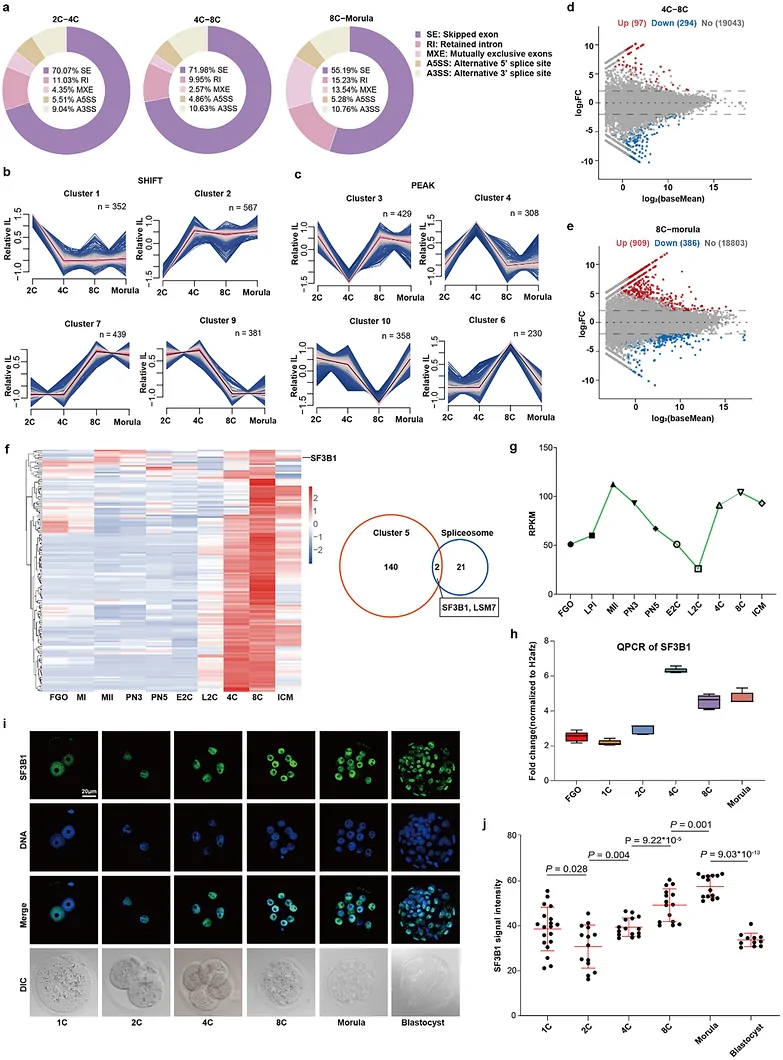
Stage‐specific alternative splicing programs reveal SF3B1 as a key regulator in early mouse embryogenesis. (a) Pie chart depicting the relative proportions of alternative splicing event (ASE) types—mutually exclusive exons (MXE), skipped exon (SE), alternative 3'splice site (A3SS), alternative 5'splice site (A5SS), and retained intron (RI)—detected across mouse pre‐implantation stages: from 2‐cell to 4‐cell, 4‐cell to 8‐cell, and 8‐cell to Morula (FDR < 0.05). (b) Representative examples of the three major PSI (percent spliced‐in) trajectory clusters—Peak, Shift, and Other—identified by Mfuzz at mouse 4‐cell and 8‐cell stages. (d,e) Scatter plots of gene‐level changes across 4‐ to 8‐cell and 8‐cell to morula transitions. Gray dots indicate non‐significant genes; red dots mark up‐regulated genes, blue dots mark down‐regulated genes. The y‐axis displays log_2_(baseMean) expression level. Significance criteria: FDR <0.05 and absolute |(log_2_FC)| ≥ 2 (FC, fold change). (f) Heatmap represents expression for Cluster 5 dynamics of splicing factors (n = 140) that are sharply up‐regulated from the 4‐cell stage onward. Venn diagram depicting their overlap with the published 21 pluripotency‐associated splicing set; SF3B1 and Lsm2 (highlighted) are the two shared regulators. (g) A Line plots showing the mRNA profiles of SF3B1 various developmental stages were examined by RNA‐seq. (h) Quantitative PCR was performed to analyze the relative expression levels of Sf3b1 in embryos at different developmental stages. Five independent biological replicates were included in the experiment, with H2afz serving as the reference gene. In the boxplots, the center line represents the median, while the upper and lower edges of the boxes indicate the upper and lower quartiles, respectively. (i,j) Representative immunostaining images (i) and quantitative analysis (j) of mouse embryos at different stages stained with anti‐SF3B1 (Green) antibody (n ≥ 10 oocytes/embryos per group). Groups were compared by two‐tailed unpaired *t*‐test. Nuclei were stained with DAPI (light blue). Scale bar: 20 µm.

To systematically profile alternative splicing across the totipotent to pluripotent transition, we re‐analyzed publicly available RNA‐seq data sets from early embryo development [28, 29], and quantified all major alternative splicing events (ASEs) by rMATS [30]. The proportion of distinct splicing isoforms was quantified using the exon inclusion level (IncLevel), with its value spanning the range of −1 to 1. In addition, IncLevelDifference (ILD) was employed to characterize the divergence in exon inclusion levels between the two samples [31].In total, this allows the detection 2068, 1749, and 2082 highconfidence ASEs in the 2‐ to 4‐cell, 4‐ to 8‐cell and 8‐cell to morula transitions, respectively (Figure S1a). Across all stages, skipped exon (SE) constituted the dominant class, accounting for 70.07%, 71.89%, and 55.19% of the events, followed by retained intron (RI), mutually exclusive exon (MXE), and alternative 5′/3′ splice‐site usage (A5SS or A3SS) (Figure 1a). Moreover, a striking directional shift in AS remodeling emerges between the 4‐to 8‐cell and 8‐cell to morula transitions. The proportion of RI events rose from 9.95% to 15.23%, whereas the share of SE cases dropped from 71.98% to 55.19%; simultaneously, mutually exclusive exon (MXE) usage surged from 2.57% to 13.54%. By contrast, A5SS and A3SS events showed no obvious directional preference (Figure 1a). These data indicate that, at this stage, the embryonic AS program selectively promotes intron retention and mutually exclusive exon inclusion while curbing exon skipping, a directional signature that parallels, yet remains clearly distinct from, the bias documented during zygotic genome activation (ZGA) [32].

To evaluate the effects of inclusion or exclusion of each alternatively spliced sequence on open reading frames (ORFs), we performed functional prediction analysis using the R package SpliceImpactR. All ASEs were classified into six functional categories: Match, protein_coding, FrameShift, Rescue, onePC, and noPC (Figure S2a–c). We performed Gene Ontology (GO) enrichment analysis on splicing events annotated as FrameShift, onePC and noPC (Figure S2d–f) and found that the corresponding genes were mainly enriched in biological processes including RNA splicing, DNA recombination, DNA double‐strand break repair, RNA modification, and protein‐RNA complex assembly. The AS remodeling expands the repertoire of protein isoforms, which may prime the embryo for subsequent lineage diversification.

Since the dynamic association between variations in differentially expressed genes (DEGs) and AS remains unclear, we profiled DEGs across these developmental transition stages (Figures 1d–e and S1b). In particular, our results demonstrated that the majority of genes involved in ASEs did not exhibit differential regulation during these stage transitions (Figure S1c), indicating that ASEs alterations and differential gene expression occur largely independently of each other; similar effects were observed in zebrafish [33]. Given that extensive transcriptional reprogramming occurs during these stages, these findings suggest that transcription isoforms are partially replaced by novel isoforms without significantly altering the overall steady‐state level of mRNA.

### 2.2 Shift‐Like Pattern Dominates AS Dynamics in 4‐/8‐cell Embryos

To dissect the temporal dynamics of AS, we applied Mfuzz to cluster cassette exons that exhibit synchronized inclusion level trajectories from the 2‐cell to morula stage. We classified them into 11 categories (Figure S1d). This analysis revealed distinct and highly coordinated patterns of splicing regulation. A subset of clusters displayed a shift‐like pattern, in which exon inclusion or skipping underwent a pronounced transition at a specific developmental stage and failed to return to the initial level, as observed in Clusters 1 and 2 at the 4‐cell stage and in Clusters 7 and 9 at the 8‐cell stage (Figure 1b). In contrast, some clusters exhibited a peak‐like pattern, characterized by a transient spike or drop in inclusion at one stage followed by a return to baseline levels, exemplified by Clusters 3 and 4 at the 4‐cell stage and Clusters 10 and 6 at the 8‐cell stage (Figure 1c). We quantified the relative contribution of each alternative splicing category and found that SHIFT dominates the repertoire of regulated exons (41.7%) compared with the other two groups (Figure S1e). These findings indicate that early embryonic development is accompanied by both sustained and stage specific modes of alternative splicing regulation, with ASEs undergoing sharp transitions particularly around the 4‐cell and 8‐cell stages that are liable to transiently disrupt protein function.

### 2.3 SF3B1 May be a Critical Splicing Factor in Early Embryonic Development Stages

To systematically identify the role of spliceosome components that underlie the emergence of embryo pluripotency, a collection of candidate splicing factors (SFs) was identified from UniProt (https://www.uniprot.org/) by retrieving mouse entries annotated with the GO term “mRNA splicing, via spliceosome” (GO:0000398). The SFs collection was subjected with previously reported RNA‐seq profiles [34] cross embryonic stages covering oocyte meiosis (FGO, MI, MII) and pre‐implantation embryogenesis (PN3, PN5, E2C, L2C, 4C, 8C, ICM) to unsupervised hierarchical clustering. This procedure resolved five expression clusters whose temporal trajectories precisely map the maternal to zygotic transition (MZT) and lineage specification window (Table S1).

Cluster 4–5 already acquire a transcriptional advantage before oocyte maturation and ZGA, suggesting that those encoded proteins may act during meiotic resumption and the MZT window (Figure S3a,c,d). Cluster 1–3 splicing factors rise in a stepwise manner after the late 2‐cell stage: cluster 1 remains persistently high at the 8‐cell and ICM stages; cluster 2 displays a bimodal peak at late 2‐cell and 4‐cell, coinciding precisely with maternal RNA clearance and the first surge of zygotic transcript. Cluster 3 is abruptly up‐regulated after the 4‐cell stage, implying that its splicing factors orchestrate dynamic spliceosomal remodeling that drives the totipotency‐to‐pluripotency cell transition for underpins early lineage commitment (Figure S3a).

After retaining transcripts confidently expressed (maximum RPKM > 20 in at least one stage), we identified 142 genes within cluster 3 that are predominantly up‐regulated at the 4‐ and 8‐cell stages (Figure 1f). To pinpoint splicing factors that drive the molecular transition from pluripotency to totipotency, we intersected the AS signature with the 23 splicing regulators previously implicated in activating totipotent marker genes; this yielded the canonical factors SF3B1 and LSM7 (Figure 1f) [19]. We therefor performed comparative profiling of SF3b complex FPKM values [20], and found that SF3B1 and SF3B6 are highly expressed and exhibit a specific peak at the 4‐cell stage relative to other candidate genes, particularly during the period from L2C to the 8‐cell stage (Figure S3b). The transcript level of SF3B1 continuously decreases from the 8‐cell stage onward, lasting until the blastocyst stage (Figure 1g). Consistent with the RNA sequencing results, qPCR analysis showed a similar expression pattern of SF3B1 in preimplantation embryos (Figure 1h). Immunofluorescence staining confirmed a nuclear localization of SF3B1 protein in embryos of different developmental stages, with an increase in SF3B1 intensity after fertilization (Figure 1i,j). These findings imply that SF3B1 may play a critical role during early embryonic development.

### 2.4 SF3B1 Dysfunction Causes Pre‐Blastocyst Developmental Defects

**FIGURE 2:**
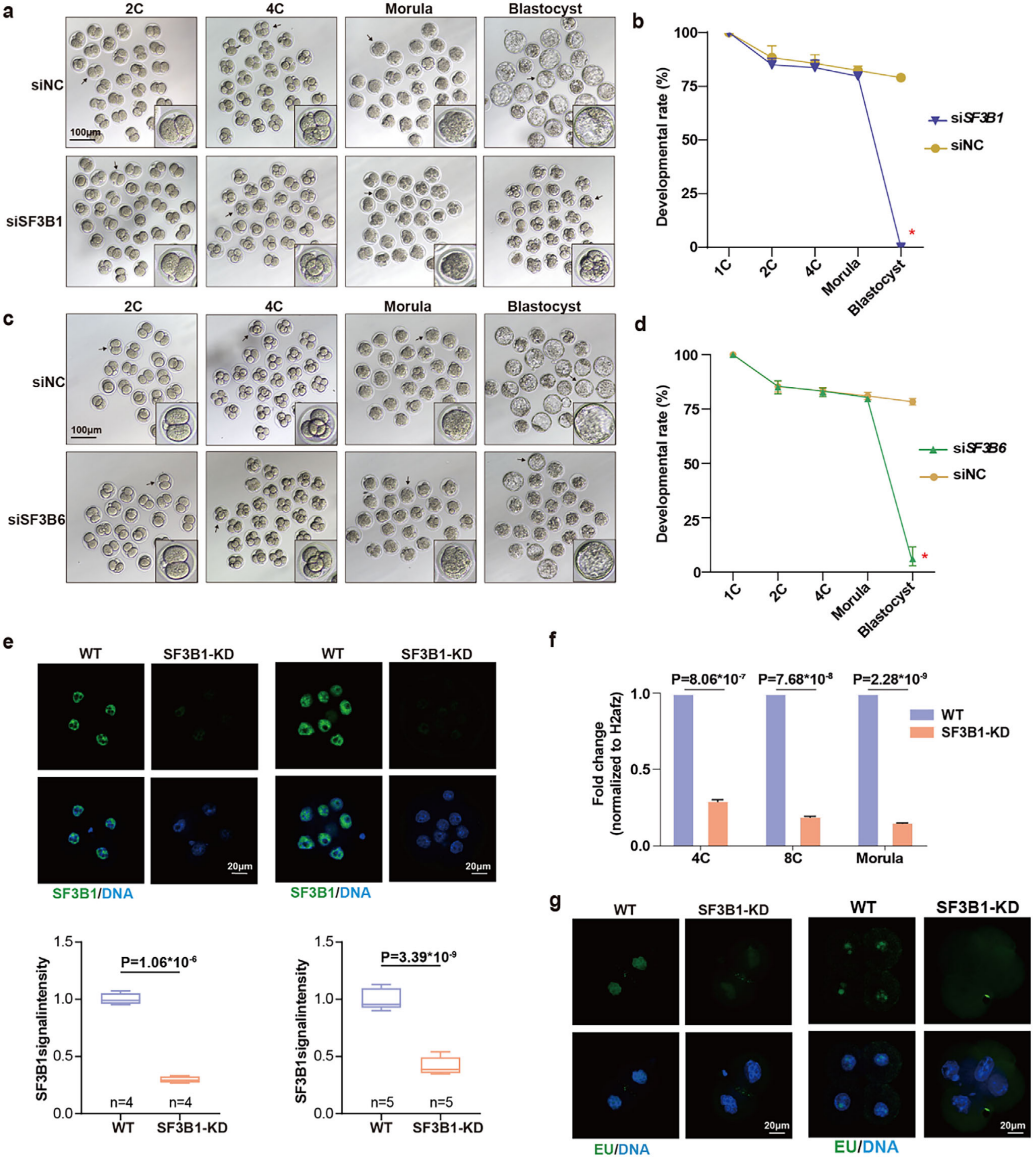
SF3B1 plays critical roles in preimplantation development. (a) Representative image represents embryonic development from the 2‐cell stage to the blastocyst stage. Mouse embryos at zygotic stage were injected with siRNA targeting SF3B1, while scrambled siRNA (siNC) served as the control. Scale bar: 100 µm. The total number of embryos in each group of treatment were as follows: siNC embryos (n = 100), siSF3B1 (n = 100). (b) Statistical evaluation of siSF3B1 injection on embryonic developmental rate. n = 3 biologically independent replicates. Data are mean ± SD. Two‐tailed Mann–Whitney U test, *p* = 0.05. (c,d) Same analyses as in panels (a,b), but performed with embryos injected with siRNA targeting SF3B6 instead of SF3B1. (e) Immunofluorescent staining images for SF3B1 in 4‐ and 8‐cell embryos after siNC or siSF3B1 microinjection (three independent experiments). Scale bar: 20 µm. Relative SF3B1 intensity was quantified with ImageJ and is expressed as mean ± SEM; groups were compared by two‐tailed unpaired *t*‐test. (f) Quantitative PCR analysis of *Sf3b1* expression at 4‐cell, 8‐cell, and morula stages. Transcript levels were normalized to endogenous *H2afz* and presented as mean ± SD of three biological replicates. (g) Immunofluorescent staining of 5‐ethynyl uridine (EU) showing RNA transcription in 2‐cell and 4‐cell embryos treated with siSF3B1.

To mechanistically dissect the roles of SF3B1 and SF3B6 in embryonic development, we designed and synthesized three siRNAs targeting each gene. Either a negative control siRNA (siNC) or SF3B1/SF3B6‐targeting siRNAs (siSF3B1/siSF3B6) were microinjected into zygote‐stage embryos and subsequently cultured in vitro to the blastocyst stage (Figure S4a). The efficiency of SF3B1 knockdown at mRNA levels was verified by RT‐qPCR. We observed a reduction in SF3B1 expression relative to that in the siNC group (Figure 2f). Moreover, immunofluorescence revealed that the protein levels of SF3B1 were markedly reduced in SF3B1‐KD embryos, while remaining comparable in NC‐KD embryos (Figures 2e and S4c). siRNA‐mediated depletion of SF3B1 at the zygote stage significantly delayed embryonic cleavage, resulting in a complete loss of morula stage formation, in contrast to control embryos, which progressed normally to the blastocyst stage (Figure 2a,b). Microinjection of SF3B6‐targeting siRNAs yielded comparable phenotypic characteristics (Figure 2c,d). However, when siRNAs (siNC or siSF3B1) were co‐injected with mCherry mRNAs into a single blastomere at the 2‐cell stage, we observed neither impaired blastomere proliferation nor abnormal blastocyst formation in the siSF3B1 group (Figure S4 h). Importantly, assessment of 5‐ethynyl uridine (EU) signals in control and siSF3B1 embryos revealed that SF3B1 depletion indeed resulted in a reduction of newly synthesized transcripts (Figure 2g).

In parallel, we employed a splicing inhibitor to pharmacologically suppress SF3B1 activity. Pladienolide B (PlaB), a competitive inhibitor SF3B complex, was added to the culture medium of embryos at the 1‐cell, 2‐cell, 4‐cell, and morula stages, with DMSO serving as the negative control (Figure S4b). Consistent with the siSF3B1 group, treatment with PlaB also adversely affected early embryonic development. Treatment at the 1‐cell stage caused developmental arrest at the 2‐cell stage, in accordance with previous research accounts [32](Figure S4d). Compared with controls, embryos treated at the 2‐cell stage rarely progressed to the 4‐cell stage (Figure S4e), with developmental arrest predominantly occurring during the 2‐ to 4‐cell transition. These findings indicate that perturbing spliceosomal components prevents ZGA. Embryos treated at either the 4‐cell or morula stage exhibited a reduced capacity to progress to the blastocyst stage (Figure S4f,g). Taken together, these findings indicate that, in addition to their critical role in driving the robust transcriptional activation during ZGA, splicing factors are also essential for proper cleavage progression beyond the 4‐cell stage.

### 2.5 SF3B1 Depletion Elicits an Aberrant Transcriptional Profile in Embryos

**FIGURE 3:**
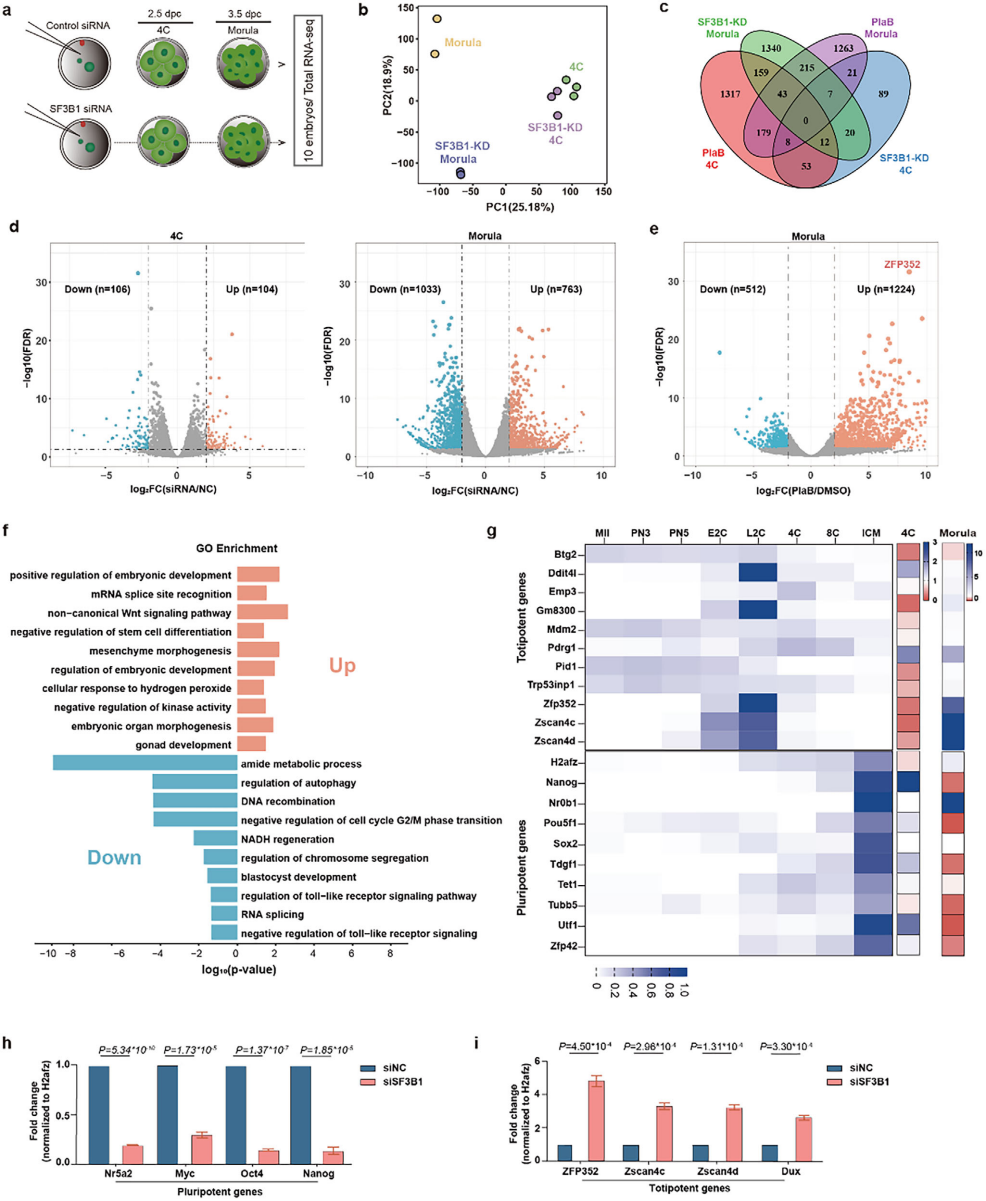
Disruption of SF3B1 leads to abnormal balance of pluripotency and totipotency gene expression in mouse 4‐cell and morula embryos. (a) Diagram illustrating the procedure of acquiring samples for total RNA‐seq analysis in embryos subjected to SF3B1‐KD and control conditions. (b) Bidimensional principal component (PC) analysis was conducted to assess the gene expression patterns in embryos undergoing preimplantation development under control and SF3B1‐KD conditions. (c) Venn diagrams comparing differentially expressed genes (DEGs) (FDR < 0.05 and |log_2_FC| ≥ 2) among different treatments. (d) Volcano plots of SF3B1‐KD versus control embryos at 4‐cell and morula: dots above the horizontal dashed line indicate DEGs (FDR ≤ 0.05, |log2FC| ≥ 2). FDR‐corrected with the Benjamini–Hochberg method (DESeq2). (e) Volcano plots showing the DEGs in morula‐stage embryos treated with plaB versus DMSO. Up‐regulated genes (red): n = 1224; down‐regulated genes (blue): n = 512. The highlighted gene Zfp352 (log_2_FC ≈ 8.48) is indicated. f GO enrichment analysis of differentially expressed genes in morula‐stage embryos upon SF3B1‐KD. Bars represent the –log_10_(*p‐*value) of the top enriched biological‐process terms; orange: up‐regulated genes, blue: down‐regulated genes. (g) Heat map showing the selected totipotency and pluripotency genes of RNA‐seq‐derived relative expression (log_2_‐normalized) from MII oocyte to ICM stage. Adjacent bubble chart indicates genes altered in 4‐cell and morula SF3B1‐KD embryos. (h,i) qPCR quantification of pluripotency (*Nr5a2*, *Myc*, *Oct4*, *Nanog*) (h) and totipotency (*Zscan4d*, *Zscan4c*, *Zfp352*, *Dux*) (i) transcript levels in SF3B1‐KD morula. Data are the mean ± SEM of three biologically independent replicates, normalized to endogenous *H2afz*.

Because SF3B1 and SF3B6 are components of the same spliceosomal complex, we performed bulk RNA‐seq on SF3B1‐KD embryos versus controls to dissect the transcriptional basis of the developmental arrest (Figure 3a). A strong correlation was observed among three biological replicates of RNA‐seq samples from each group (Figure S5a), thus confirming the precision of the acquired gene expression profiles (Figure 3b). We selected mRNAs possessing dependable sequence annotations and fragments per kilobase of transcript per million mapped reads (FPKM> 1) for examined the DEGs. Compared to the control group, in the 4‐cell stage embryos, the SF3B1‐KD group exhibited 106 upregulated genes and 104 downregulated genes (FDR ≤ 0.05; |log2(FC)| ≥ 2). Notably, at the morula stage, a total of 1796 genes were differentially expressed, with 1033 upregulated and 763 downregulated genes (Figure 3d and Table S3).

**FIGURE 4:**
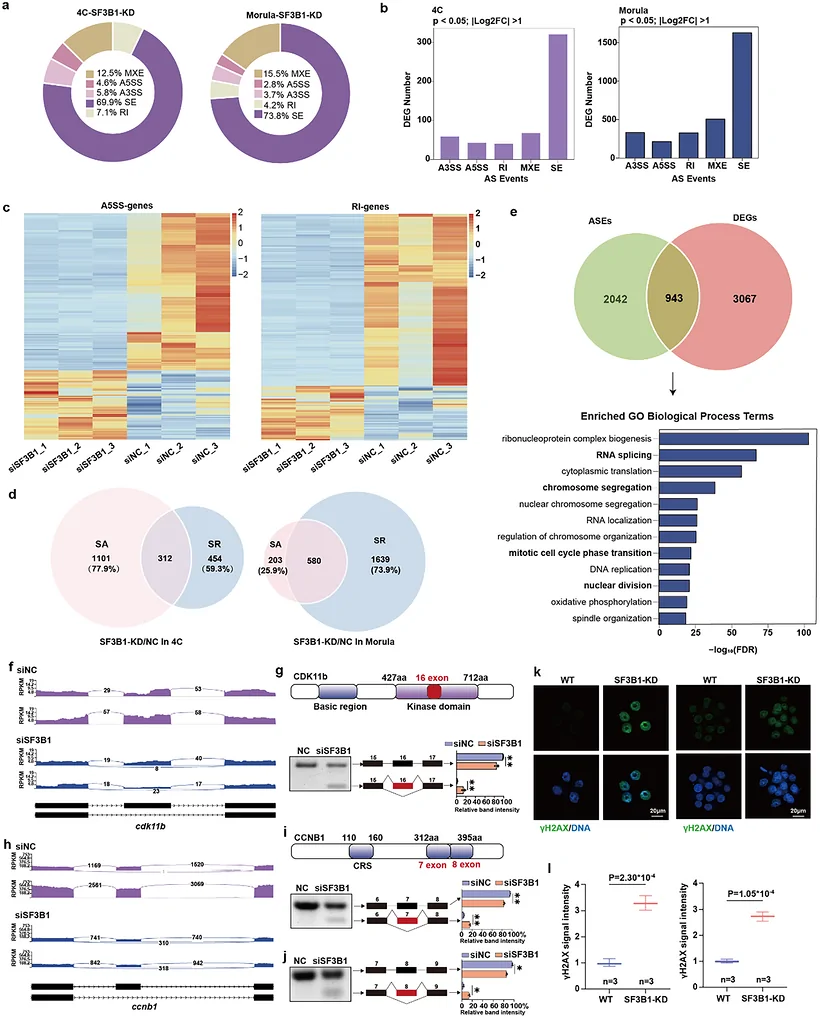
SF3B1 directly shapes the alternative splicing landscape governing the cell cycle. (a) Proportions of five ASE categories‐MXE, SE, A3SS, A5SS, and RI‐significantly shifted in SF3B1‐KD versus control embryos at the 4‐cell and morula stages. (b) Counts of DEGs (*p* < 0.05; |log_2_FC| > 1) corresponding to each AS event type. (c) Heatmap displaying the normalized expression patterns of transcripts A5SS and RI events at morula stage embryos. Values represent normalized relative mRNA abundance. (d) Venn diagrams showing the relationship of categorized as splicing active (SA) and splicing repressive (SR) between SF3B1‐KD and control embryos at the 4‐cell (left) and morula (right) stages. (e) A Venn diagram showing the overlap between ASEs and DEGs in siSF3B1 versus siNC (top). GO analysis of the 943 shared mRNAs is presented (bottom). (f) Sashimi plots depict read coverage and exon–exon junction usage for *cdk11b*. (g) Diagram of CDK11b protein architecture highlighting the kinase domain and basic region. Semiquantitative RT‐PCR (*n* = 3 per group) mapped *cdk11b* ectopic splicing in SF3B1‐KD morula, and representative gels combined with pooled quantification of the specified fragment are showed accordingly. (h) Same as panel f, but for *ccnb1*. (i,j) The ectopic splicing of *ccnb1* by semiquantitative RT‐PCR. (k) Immunofluorescent staining of γH2AX showing DNA double‐strand breaks (DSBs) in 2‐cell and 4‐cell embryos treated with siSF3B1. Relative signal intensity was quantified with ImageJ and is expressed as mean ± SEM; groups were compared by two‐tailed unpaired *t*‐test.

To further identify the PlaB treatment for DEGs, we performed bulk RNA‐seq on PlaB treatment on 4‐cell and morula embryos vs. controls to dissect the transcriptional basis of the developmental arrest. A strong correlation was observed among three biological replicates of RNA‐seq samples from each group (Figure S5b–d). Compared to the control group, in the 4‐cell stage embryos, the PlaB group exhibited 881 upregulated genes and 890 downregulated genes (FDR ≤ 0.05; |log2(FC)| ≥ 2) (Figure S5e). In contrast, at the morula stage, a total of 1756 genes were differentially expressed, with 1244 upregulated and 512 downregulated genes (Figure 3e and Table S3). Differentially DEGs exhibited stage‐ and treatment‐specific patterns. Morula SF3B1‐KD and PlaB‐treated shared 265 DEGs, 4‐cell and morula SF3B1‐KD shared 39 DEGs (Figure 3c). Pearson correlation analysis revealed a marked global expression correlation (R = 0.869) between SF3B1‐KD and PlaB‐treated morula embryos (Figure S5f). To further investigate the function of DEGs, we performed GO analysis on SF3B1‐KD samples. The DNA damage response (DDR) pathway is significantly upregulated in 4‐cell embryos, consistent with the γH2AX immunostaining pattern, which reflects the level of DNA double‐strand breaks (Figures 4k and S5 g). Notably, the downregulated genes in morula were associated with metabolic and autophagy pathways, genome maintenance (including DNA recombination), mitotic precision (G2/M cell‐cycle control, chromosome segregation), and blastocyst development essential for early embryogenesis (Figure 3f). In contrast, the upregulated genes were enriched for pathways involved in splice site recognition, non‐canonical Wnt signaling, and embryonic developmental regulatory networks that govern cell fate transitions (Figure 3f). Only SF3B1 was markedly downregulated in SF3B1‐KD embryos, while other SF3B family genes remained unchanged. PlaB induced only slight upregulation of PHF5A. Therefore, global transcriptomic alterations upon SF3B1 depletion are not due to secondary dysregulation of other SF3B complex members (Figure S6d–f). Together, our results suggest that SF3B1 plays a critical role in modulating gene expression involved in embryonic development.

### 2.6 SF3B1 is Required for the Conversion From Totipotency to Pluripotency

Building on a SPAR‐seq survey showing 52 conserved AS events governing ES cell pluripotency, neural differentiation and somatic reprogramming upon KD of 1536 genes [34]. Given its role as a core AS factor, we propose that SF3B1 mediates the transition from totipotency to pluripotency. To test this, we mapped the marker gene landscape at the 4‐cell and morula stages. As expected, totipotent genes (*Btg2*, *Ddit4l*, *Emp3*, *Gm8300*, *Mdm2*, *Pdrg1*, *Pid1*, *Trp53inp1*, *Zfp352*, *Zscan4c*, *Zscan4d*) displayed peak expression at the 2‐cell and 4‐cell stages, declined abruptly upon SF3B1 KD in the morula (Figure 3g). In contrast, core pluripotent genes (*H2afz*, *Nanog*, *Nr0b1*, *Pou5f1*, *Sox2*, *Tdgf1*, *Tet1*, *Tubb5*, *Utf1*, *Zfp42*) normally rise from the 8‐cell stage through the ICM, yet this developmental upregulation was abolished in SF3B1‐KD morula embryos (Figure 3g). Notably, a similar dysregulation of genes associated with the transition from totipotency to pluripotency was observed in PlaB‐treated morula embryos (Figure S6c). Consistently, RT‐qPCR analysis of pluripotent regulators (*Nr5a2*, *Myc*, *Oct4*, and *Nanog*) and totipotent genes (*ZFP352*, *Zscan4c*, *Zscan4d*, and *Dux*) confirmed that these developmentally stage‐specific regulators were markedly dysregulated upon SF3B1 knockdown (Figure 3h,i).

Early mouse embryonic development primarily depends on a gene regulatory network, with transcription factors (TFs) serving as key components [35]. At 4‐cells, SF3B1 inhibition (SF3B1‐KD or PlaB treatment) down‐regulated *Zfp352*, *Tcstv3*, and *Tcstv1*, while up‐regulating *Esrrb*, *Tfcp2l1*, *Gata6* and *Tbx3* (Figure S7a). In morula, the response reversed: ZGA‐specific transcripts (*Zscan4d*, *Zfp352*, *Tcstv1*/3, *Zc3h12a*) were selectively lost (log_2_FC ≈ −2 to −4) due to long‐intron splicing failure, whereas canonical pluripotency TFs (*Pou5f1*, *Nanog*, *Myc*, *Tfcp2l1*, *Esrrb*) and early‐lineage determinants (*Gata3*, *Cdx2*, *Klf5/8*) accumulated (log_2_FC ≈ +2 to +5) (Figure S7b). Concurrent down‐regulation of Dnmt3b/Uhrf1 may create a low‐methylation window that further amplifies TF rewiring (Figure S7b). SF3B1 loss triggers a “transcriptional runaway” signature: minor pluripotency network fluctuations at the 4‐cell stage are followed by a dramatic rewiring of core pluripotency TFs in the morula, which derails lineage commitment and arrests embryonic development. Furthermore, we observed a substantial reduced expression of tight junction genes during morula stage, indicating a specific collapse of junctional integrity upon splicing blocked induced transcriptional overshoot (Figure S7c). In contrast, the Wnt module and key stem cell differentiation regulators are coordinately up‐regulated upon SF3B1 loss in morula (Figure S7d, e). Notably, we found pharmacological inhibition of Wnt signaling with IWR‐1 or IWP‐2 partially rescues the blastocyst developmental arrest induced by SF3B1‐KD and PlaB‐treated embryos (Figure S7f,g), indicating that aberrant Wnt activation is a key contributor of developmental defects induced by SF3B1 dysfunction.

We propose that SF3B1 depletion at the morula stage reprograms the TF circuitry governing the totipotency‐to‐pluripotency transition, while simultaneously disrupting tight junction assembly and elevating Wnt core components together with stem cell exit regulators. These combined defects constitute a major barrier preventing progression to the blastocyst stage.

### 2.7 SF3B1 Deficiency Perturbs AS of Mitotic Cell Cycle Regulators and Induces DNA Damage

By compared the genome wide AS perturbations of SF3B1‐KD and control sample, it is possible to reveal regulatory potential of SF3B1 in embryogenesis. Notably, enriched SE accounted for 69.9% and 73.8% at these changes, indicating that SE is the predominant driver of splicing dysregulation at both stages (Figure 4a).

To systematically assess how SF3B1‐driven AS reshapes the transcriptome, we analyzed significant DEGs within alterations major ASEs. Further statistical quantified DEG within SEs again constituted the largest fraction of ASEs in both groups (Figure 4b). Transcripts carrying A5SS or RI events showed the predominantly down‐regulation in morula relative to those with MXE, SE or A3SS event (Figures 4c and S8a), whereas at the 4‐cell stage the ratio of down‐regulated genes was highest within A3SS compared with A5SS and MXE (Figures 4c and S8b). Thus, SF3B1 depletion does cause a global AS perturbations disrupting embryonic development [36]. Further, we categorized splicing changes as either activation (SA) or repression (SR) (Figure S8c) [37]. SF3B1‐KD skews AS in a stage specific manner: at the 4‐cell stage 1413 events are enhanced versus suppressed 766 (SA/SR ≈ 1.84), whereas at morula 783 events are enhanced versus 2219 suppressed (SA/SR ≈ 0.35) (Figure 4d). SF3B1 depletion in morula concurrently rewired the transcriptome and the spliceome, yielding 3,067 differential ASEs and 2042 DEGs (Figure 4e).

To further verify the role of SF3B1‐mediated AS perturbation, we performed GO enrichment analysis on AS‐coupled DEGs, which revealed significant terms primarily involved in pathways associated with RNA‐splicing itself, followed by chromosome segregation, mitotic phase transition, DNA replication, and spindle organization (Figure 4e). The results highlight SF3B1 loss disrupts co‐transcriptional splicing programs essential for maintaining mitotic fidelity during early embryogenesis. Consistent with this, we observed pronounced splicing dysregulation in key mitotic regulators, including *Ccnb1* (G2/mitotic‐specific cyclin‐B1) and *Cdk11b*, both of which exhibited aberrant exon usage upon SF3B1 depletion. Skipping of exon 16 in Cdk11b is expected to compromise its kinase activity (Figure 4f,g), leading to insufficient phosphorylation of Pol II CTD‐Ser2 [38] (Figure S7k) and decrease centromeric transcription (Figure S7l). Disruption of CDK11B exhibited mitotic arrest and genomic instability in blastocyst cells, ultimately triggering apoptosis and early embryonic lethality [39]. In line with this model, immunofluorescence staining of α‐Tubulin and γH2AX showed aberrant spindle assembly and increased DNA damage in PlaB‐treated embryos (Figure S7i,j). Consistently, TUNEL staining detected markedly elevated apoptotic signals in SF3B1‐KD blastocysts relative to control embryos (Figure S7 h). While skipping of exons 7 or 8 in *Ccnb1*(Figure 4h–j), a core regulator of G2/M cell cycle transition, likely disrupts its C‐terminal domain and thereby impairs mouse embryonic development (Figure S9g,h). Aberrant alternative splicing was also detected in other key regulators of mouse early embryonic development, such as exons 11 in *Mdm2* (Figure S9a,b) which safeguards blastocyst formation by ubiquitinating p53 to suppress apoptosis and maintain cell cycle progression, and exons 18 in *Ezh2* (Figure S9c,d), which maybe deposits H3K27me3 to silence TE lineage genes. Defective checkpoint enforcement is known to induce replication stress, which in turn leads to replication fork collapse and accumulation of DNA double‐strand breaks (DSB). Accordingly, we found that SF3B1 depletion impaired DNA damage response, resulting in elevated γ‐H2AX foci accumulation and compromised DNA integrity (Figure 4k), consist with previous evidence that splicing dysfunction contributes to DNA damage [10]. These data suggestion that SF3B1 regulate the splicing patterns associated with the cell cycle.

### 2.8 Binding Landscape of SF3B1 in Mouse Embryos

**FIGURE 5:**
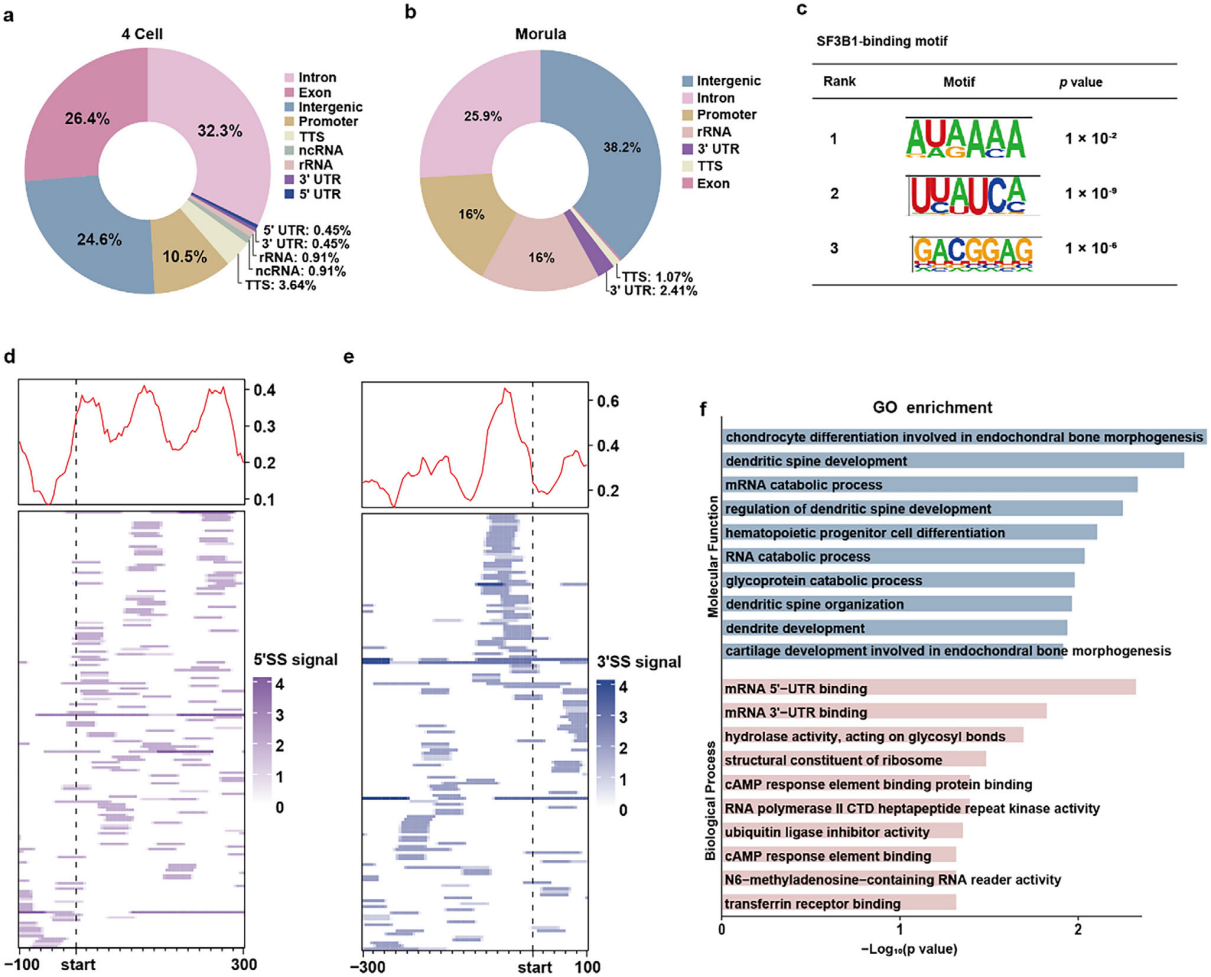
The global landscape of SF3B1‐binding sites. (a) Venn diagram showing genomic distribution of the SF3B1‐binding reads in 4‐cell. (b) Venn diagram showing genomic distribution of the SF3B1‐binding reads in morula. (c) Enriched SF3B1‐binding motifs identified by LACE‐seq and their *p* values are shown. (d,e) The signal intensity distributions of 5’ splicing site of SF3B1 binding in morula (d) and 3’ splicing site (e). f GO enrichment analysis of SF3B1‐specific targets in morula. The represent results from two independent experiments.

To elucidate the underlying mechanisms, we performed transcriptome‐wide analyses of SF3B1 binding. We performed LACE‐seq (the linear amplification of complementary DNA ends and sequencing) [40] to globally profile SF3B1 RNA‐binding sites in 4‐cell and morula embryos, and observed that intronic regions are the predominant targets of SF3B1‐RNA cross‐linking sites across distinct developmental stages (Figure 5a,b). SF3B1‐binding peaks in mouse embryos were significantly enriched in AU‐rich consensus motifs (Figure 5c), likely reflecting the U‐rich intronic regions are a common feature surrounding branch point sequences (BPS) [41] that facilitate spliceosome assembly. We further performed RT‐qPCR to verify intronic RNA enrichment of *Elf2* in SF3B1 LACE‐seq samples (Figure S9i).

Metagene analysis revealed that SF3B1 binding signals were strongly enriched upstream of the 3′ splice site, peaking within the canonical branch point region, whereas no comparable enrichment was observed around the 5′ splice site (Figure 5d,e). These results are consistent with the established role of SF3B1 as a core component of the U2 snRNP involved in branch point recognition during early spliceosome assembly. GO analysis of SF3B1 bound and regulated genes revealed significant enrichment in RNA metabolic processes, including mRNA catabolism, ribosome biogenesis, and translational machinery (Figure 5f). Cellular component terms were dominated by ribosomal and cytosolic ribosome‐associated categories, consistent with the central role of SF3B1 in pre‐mRNA splicing and post‐transcriptional gene regulation.

### 2.9 SF3B1^IDR^ Exhibits an Intrinsic Capacity for LLPS in Vitro and is Modulated by Crowding, Ionic Strength, and RNA

**FIGURE 6:**
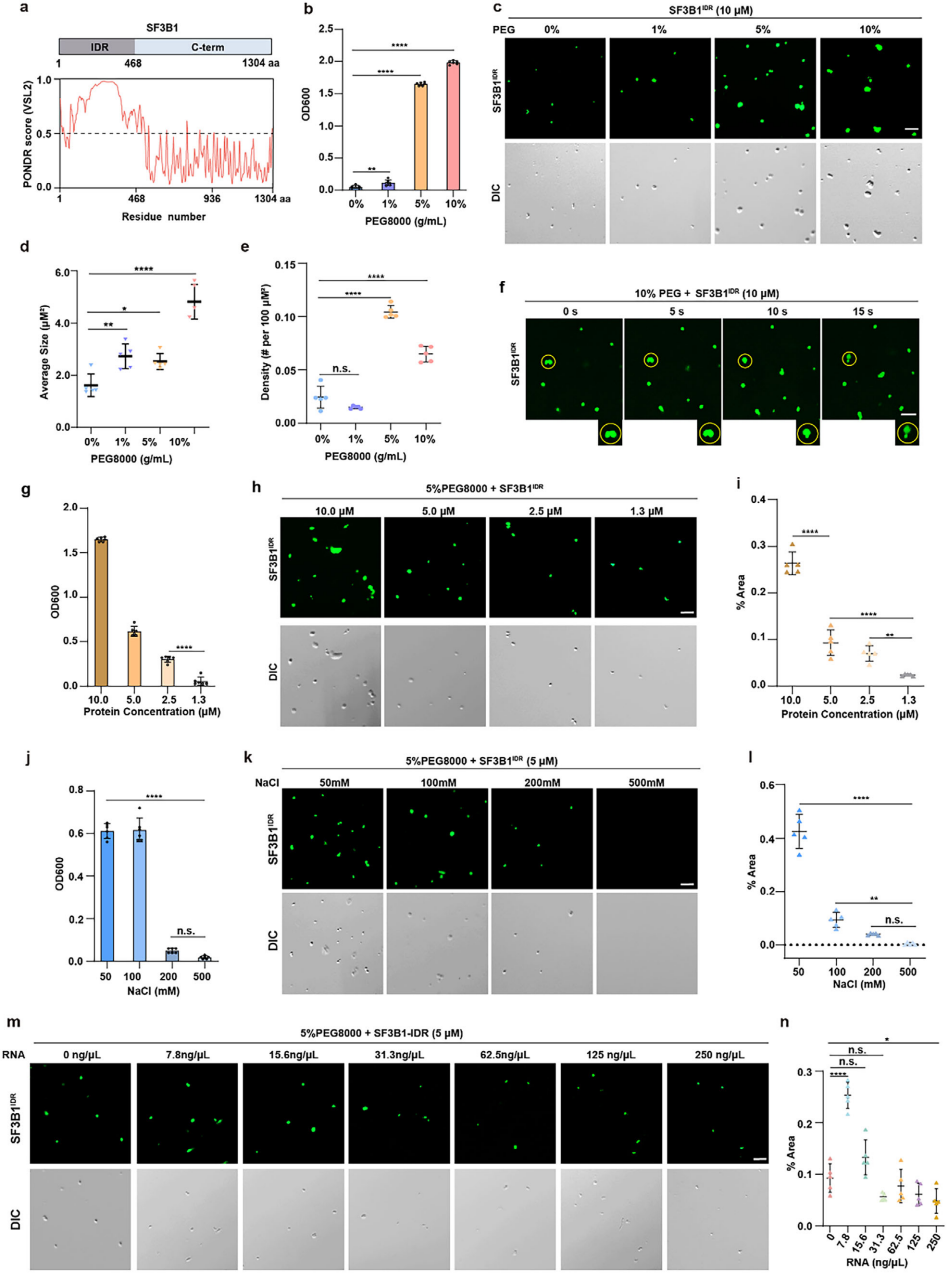
Characterization of SF3B1^IDR^ phase separation. (a) Domain structure and intrinsic disorder tendency of SF3B1. The top panel shows the domains of SF3B1, along with PONDR analysis. (b) Turbidity assays monitoring LLPS of SF3B1^IDR^ across different PEG8000 concentrations (n = 6). (c) Representative fluorescence (AF488‐labeled) and differential interference contrast (DIC) images demonstrating droplets of 10 µM SF3B1^IDR^ with varying concentrations of PEG8000. Scale bar: 10 µm. d‐e Quantitative analysis of SF3B1^IDR^ droplet average size (d) and droplet density per 100 µm^2^ area (e) under increasing PEG8000 concentrations (0%, 1%, 5% and 10% w/v). n = 5 biologically independent samples. (f) Representative time‐lapse images showing fusion events of 10 µM SF3B1^IDR^ droplets in T50N100 buffer supplemented with 10% PEG8000. Insets show magnified views of tracked droplets highlighted in yellow circles. Scale bar, 10 µm. (g) Turbidity assays monitoring LLPS of SF3B1^IDR^ across varying concentrations in T50N100 buffer supplemented with 5% PEG8000 (n = 6). (h) Representative fluorescence (AF488‐labeled) and DIC images demonstrating SF3B1^IDR^ droplet formation at varying protein concentrations in T50N100 buffer supplemented with 5% PEG8000. Scale bar: 10 µm. (i) SF3B1^IDR^ condensate coverage (% Area) at varying protein concentrations (1.3–10.0 µM). n = 5 independent replicates. (j) Turbidity assays monitoring LLPS of 5 µM SF3B1^IDR^ in 50 mM Tris‐HCl buffer (supplemented with 5% PEG8000) at different NaCl concentrations. (n = 6). (k) Representative fluorescence (AF488‐labeled) and DIC images of 5 µM SF3B1^IDR^ in 50 mM Tris‐HCl buffer with 5% PEG8000, at different NaCl concentrations (50, 100, 200, 500 mM). Scale bar: 10 µm. (l) SF3B1^IDR^ condensate coverage (% Area) in 50 mM Tris‐HCl buffer (5% PEG) across different NaCl concentrations. n = 5 independent replicates. (m) Representative images depicting the effect of total RNA on SF3B1^IDR^ condensation. SF3B1^IDR^ was mixed with different concentrations of total RNA in T25N50 buffer (25 mM Tris‐HCl, 50 mM NaCl, pH 7.5) supplemented with 5% PEG8000. Scale bar = 10 µm. (n) SF3B1^IDR^ condensate coverage (% Area) in T25N50 buffer (5% PEG8000) across different RNA concentrations (7.8–250 ng/µL). All quantitative data are presented as mean ± SD. Turbidity assays (b, g, j) used n = 6 biologically independent samples; droplet/density quantifications (d, e, i, l, n) used n = 5 biologically independent samples. Statistical differences were determined by one‐way ANOVA with Tukey's multiple comparisons test. Significance levels: n.s., not significant; *p < 0.05, **p < 0.01, ***p < 0.001, ****p < 0.0001.

RBPs enriched in intrinsically disordered regions (IDRs) frequently exhibit an intrinsic capacity for LLPS, enabling the assembly of dynamic ribonucleoprotein condensates [42]. SF3B1 is a core component of the U2 small nuclear ribonucleoprotein (U2 snRNP) and is essential for pre‐mRNA splicing [43, 44]; however, whether it possesses phase separation potential has remains unclear. To explore this possibility, we first analyzed the sequence features of SF3B1 using PONDR VL‐XT [45], which identified the N‐terminal region (residues 1–468) as intrinsically disordered (Figure 6a). Consistent with this, catGRANULE 2.0 [46] predicted high granule‐forming propensity and LLPS propensity within the N‐terminal IDR (Figure S10a). These observations prompted us to directly test whether the SF3B1 N‐terminal IDR is sufficient to support LLPS. To this end, we reconstituted the system in vitro using homogeneously purified SF3B1^IDR^ (Figure S10b). Under physiological buffer conditions (50 mM Tris‐HCl, 100 mM NaCl, pH 7.5), SF3B1^IDR^ exhibited visible turbidity (Figure S10c), indicative of macroscopic phase separation. Fluorescent labeling of SF3B1^IDR^ with Alexa Fluor 488 revealed the spontaneous formation of spherical, micrometer‐sized droplets (Figure 6c), confirming that the SF3B1^IDR^ alone is sufficient to support LLPS in vitro.

We then examined how physicochemical parameters relevant to the nuclear environment influence SF3B1^IDR^ phase behavior. Increasing concentrations of the macromolecular crowding agent PEG8000 (∼300–400 mg/mL macromolecules equivalent [47]) enhanced droplet formation in a dose‐dependent manner (Figure 6b,c). At higher PEG concentrations (5%‐10%), droplets became fewer but larger (Figure 6d,e), consistent with droplet coarsening driven by LLPS thermodynamic under crowded conditions [48]. Time‐lapse imaging further showed that SF3B1^IDR^ droplets gradually transitioned from a highly dynamic, liquid‐like state to more rigid assemblies over time (Figure 6f), a behavior commonly observed during condensate maturation [49, 50]. Consistent with a concentration‐dependent phase separation, SF3B1^IDR^ exhibited a sharp increase in phase separation above ∼2.5 µM protein (Figure 6g–i). Ionic strength strongly modulated droplet formation, with maximal LLPS observed at 50–100 mM NaCl and substantial suppression at high salt concentrations (≥200 mM) (Figure 6j–l), indicating an important contribution of electrostatic interactions to SF3B1^IDR^ assembly.

Given the RNA‐binding function of SF3B1 in pre‐mRNA splicing, we further investigated the effect of RNA on SF3B1^IDR^ phase separation. Total cellular RNA exerted a biphasic effect, enhancing droplet formation at low RNA concentrations (≤31.3 ng/µL) while progressively inhibiting LLPS at high concentrations (≥125 ng/µL) (Figure 6m,n). This behavior is consistent with RNA acting as a multivalent scaffold at low concentrations and promoting solubilization at higher levels [42, 51].

Together, these results demonstrate that the N‐terminal IDR of SF3B1possesses a robust intrinsic capacity for LLPS in vitro, with condensate formation and material propertied being finely tuned by macromolecular crowding, protein concentration, ionic strength, and RNA. These properties closely mirror the physicochemical sensitivities of the nucleus, supporting a model in which SF3B1 phase behavior is dynamically regulated to facilitate its splicing functions in cells.

### 2.10 Domain‐Resolved Phase Separation of SF3B1

**FIGURE 7:**
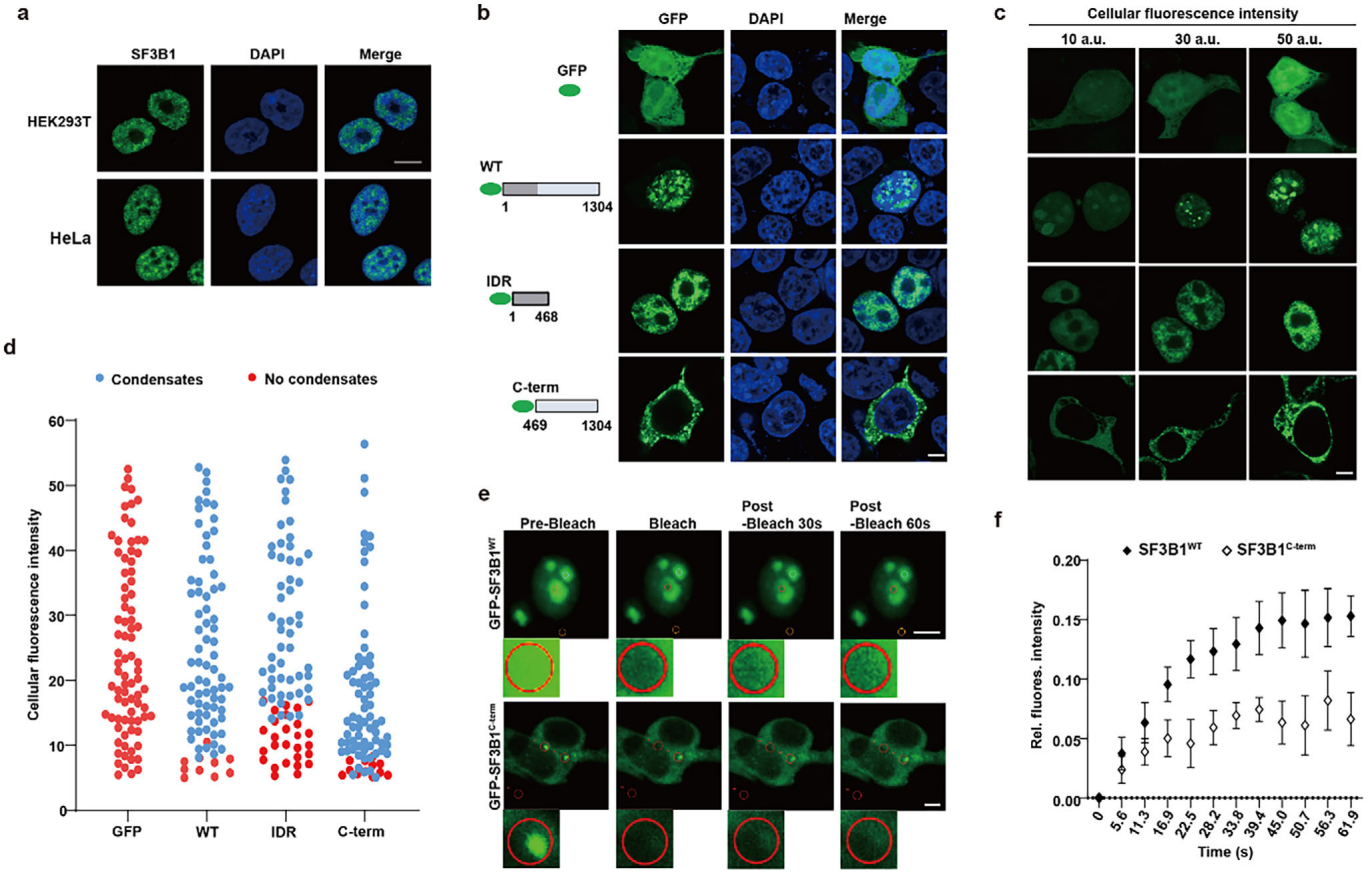
SF3B1 undergoes LLPS in a concentration dependent manner. (a) Immunofluorescence staining of endogenous SF3B1 (green) in HEK293T and HeLa cells. Nuclei were counterstained with DAPI (blue). Scale bar: 10 µm. (b) Representative confocal image of HEK293T and Hela cells transfected with different forms of recombinant GFP‐SF3B1 constructs, including GFP, GFP‐SF3B1^WT^, GFP‐SF3B1^IDR,^ and GFP‐SF3B1^C‐term^. Nuclear area visualized by Hoechst staining. Scale bar: 5 µm. (c) Representative confocal images of HEK293T cells expressing GFP, GFP‐SF3B1^WT^, GFP‐SF3B1^IDR,^ and GFP‐SF3B1^C‐term^ at 10, 30, and 50 arbitrary units (a.u.). Scale bar: 5 µm. Representative images were selected based on relevant statistical analyses from three independent experiments with similar results. (d) Quantitative phase diagram depicting the cellular fluorescence intensity of SF3B1 domains observed. Each dot represents the SF3B1 concentration measured in an individual cell. Blue dots indicate positive phase separation, while red dots indicate negative phase separation. (e) Representative images of the FRAP experiment in HEK293T cells transfected with GFP‐SF3B1^WT^ or GFP‐SF3B1^C‐term^. Images depict the Pre‐Bleach (before photobleaching), Bleach (during photobleaching), and Post‐Bleach (30s and 60s after photobleaching, relative to 0s at bleaching). The red circle highlights the organelle targeted for bleaching. Scale bar: 5 µm. (f) The quantification of FRAP data for GFP‐SF3B1^WT^ or GFP‐SF3B1^C‐term^ puncta. Time 0 refers to the time point of the photobleaching pulse. Data are presented as the means ± SEMs (n = 6), n = individual SF3B1 nuclear condensate.

To assess cellular LLPS of endogenous SF3B1, we performed immunofluorescence staining in 293T and HeLa cells using SF3B1‐specific antibody, which exhibited distinct punctate foci (Figure 7a). Fluorescence results revealed SF3B1‐enriched nuclear assemblies are classical nuclear speckles (Figure S10d–f). We next delineated the functional region that drives SF3B1 phase separation in vivo. GFP‐tagged full‐length SF3B1 (GFP‐SF3B1) and truncation constructs corresponding to the N‐terminal IDR (GFP‐SF3B1^IDR^) or the C‐terminal region (GFP‐SF3B1^469‐1304^) were expressed in HEK293T cells (Figure 7b). Confocal microscopy revealed that GFP alone was diffusely distributed throughout both the nucleus and cytoplasm. In contrast, GFP‐SF3B1 displayed nuclear localization with a combination of diffuse nucleoplasmic signal and discrete intranuclear foci, consistent with condensate formation within the nuclear compartment.GFP‐SF3B1^IDR^ similarly localized to the nucleus and formed punctate nuclear condensates, indicating that the N‐terminal IDR is sufficient to promote condensate assembly in vivo. By contrast, GFP‐SF3B1^469‐1304^ was largely excluded from the nucleus and instead accumulated in the cytoplasm, where it formed prominent cytoplasmic condensates (Figure 7b), consistent with the presence of a nuclear speckle‐targeting sequence in the N‐terminal region [52].

For all constructs, condensate formation was observed only above a defined expression threshold, quantified by GFP fluorescence intensity, below which the signal remained largely diffuse; once this threshold was exceeded, both the number and size of condensates increased with expression level (Figure 7c), consistent with the concentration‐dependent characteristic of phase separation [53, 54]. Notably, full‐length GFP‐SF3B1 and GFP‐SF3B1^469‐1304^ exhibited lower apparent concentrations thresholds for condensate formation ∼10 arbitrary units (a.u.) compared with GFP‐SF3B1^IDR^ (∼15 a.u.) (Figure 7d), suggesting that the C‐terminal structured region lowers the energetic barrier for assembly, potentially by providing additional multivalent interaction interfaces.

We next examined the dynamic properties of these condensates using fluorescence recovery after photobleaching (FRAP). GFP‐SF3B1^IDR^ condensates showed rapid fluorescence recovery, consistent with highly dynamic, liquid‐like assemblies (Video S1). In contrast, GFP‐SF3B1^1469‐1304^ condensates exhibited minimal recovery (∼5%), indicative of more rigid or gel‐like material states [50]. Full‐length SF3B1 displayed intermediate recovery kinetics (∼15%) (Figure 7e, f), suggesting that the structured C‐terminal domain modulates the dynamics of IDR‐driven condensates in the context of the full‐length protein.

Together, these results demonstrate that SF3B1 forms concentration‐dependent condensates through coordinated contributions from distinct domains. The N‐terminal IDR (localized in the nucleus) is sufficient to drive dynamic nuclear condensate formation, whereas the C‐terminal HEAT‐repeat‐containing region tunes the concentration threshold, subcellular localization, and material properties of these assemblies. This domain‐resolved organization reveals a modular mechanism by which structured and disordered regions cooperate to regulate condensate behavior, consistent with principles observed in other multi‐domain RBPs such as hnRNPA1 and FUS [55, 56].

### 2.11 Transcription and Nuclear Remodeling Driven SF3B1 Condensation is Required for Early Embryogenesis

**FIGURE 8:**
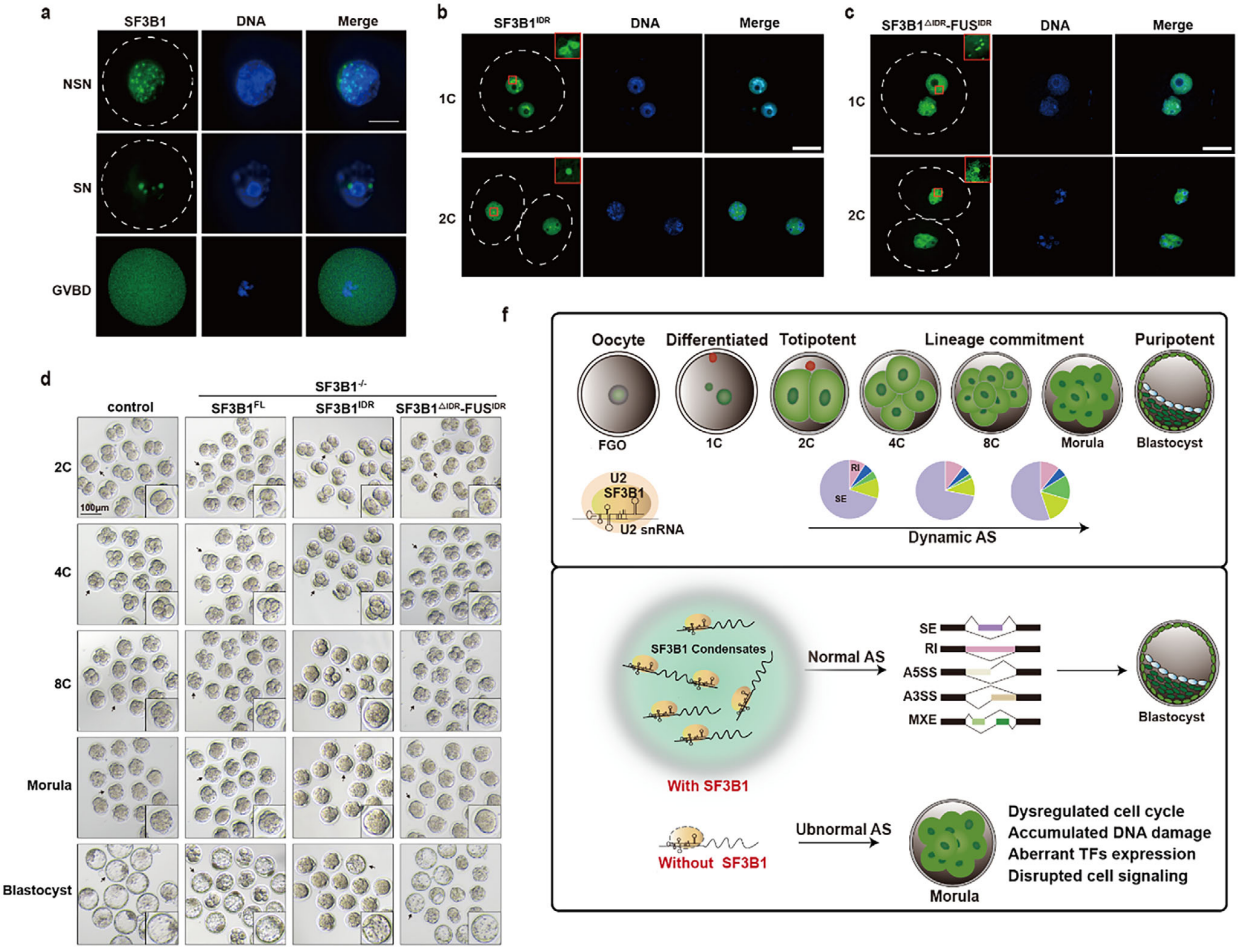
LLPS of SF3B1 is essential for early mouse embryonic development. (a) Representative immunostaining images indicate NSN, SN, and GVBD oocyte stained with anti‐SF3B1 (Green) antibody (n ≥ 5 oocytes per group). Nuclei were stained with DAPI (light blue). Scale bar: 20 µm. (b,c) Confocal images of embryos microinjected at the 1‐cell stage with GFP‐tagged mRNAs encoding GFP–SF3B1^IDR^ (b) or GFP‐SF3B1^ΔIDR−^FUS^−IDR^ (c), and imaged at zygote and 2‐cell stages. Scale bar: 20 µm. (d) Representative image represents embryonic development from the 2‐cell stage to the blastocyst stage. Mouse embryos at zygotic stage were injected with siRNA targeting SF3B1 combined with GFP‐SF3B1^FL^, GFP‐SF3B1^IDR^or GFP‐SF3B1^ΔIDR‐FUS‐IDR^. Scale bar: 100 µm. n = 3 biologically independent replicates. (g) Model of SF3B1 mediated alternative splicing in mouse early embryo development.

Upon entry into meiotic maturation, oocytes silence transcription and reprogram splicing patterns through a nuclear switch from the non‐surrounded nucleolus (NSN) to the surrounded nucleolus (SN) configuration, accompanied by chromatin compaction and cessation of nascent pre‐mRNA synthesis. In transcriptionally active NSN oocytes, SF3B1 is dispersed as numerous small speckles that support efficient spliceosome assembly (Figure 8a). In transcriptionally quiescent SN oocytes, splicing activity is reduced, and SF3B1 condenses into fewer, larger speckles, indicative of splicing inactivity. At Germinal Vesicle Breakdown (GVBD), disassembly of the nuclear envelope abolishes the nucleoplasmic boundary, allowing nuclear SF3B1 to diffuse throughout the cytoplasm in a dispersed pattern (Figure 8a). These data demonstrate that SF3B1 condensation is a transcription‐coupled event occurring exclusively within the nucleus. Immunofluorescence further confirmed that SF3B1 exhibited punctate nuclear localization at all stages of early mouse embryogenesis (Figure 1i). As development proceeds, SF3B1 protein levels gradually increase; concomitantly, the nucleus shrinks markedly after the 1‐ and 2‐cell stages, raising the nuclear‐to‐cytoplasmic ratio and compacting nuclear architecture [57]. These changes collectively render the SF3B1 speckles appear increasingly condensed (Figure 1i).

To dissect how SF3B1‐driven phase separation influences early mouse embryogenesis, we asked whether developmental arrest caused by SF3B1 depletion could be rescued by protein reintroduction. The FUS intrinsically disordered region (FUS^IDR^) is a canonical LLPS‐driving element characterized by low complexity and enrichment in glycine, serine, and tyrosine residues [58]. We therefore generated chimeric proteins SF3B1^△IDR^‐FUS^IDR^, in which the native IDR of SF3B1 was replaced with FUS^IDR^. Following microinjection SF3B1^IDR^ and SF3B1^△IDR‐FUSIDR^ mRNA into zygotes, large nuclear speckles were detected in early embryos (Figure 8b,c). To assess functional rescue, we injected SF3B1^FL^, SF3B1^IDR^, and SF3B1^△IDR^‐FUS^IDR^ into SF3B1‐KD embryos, and found that the developmental delay caused by SF3B1 deficiency can be partly rescued by injecting SF3B1^FL^ mRNA, but not SF3B1^IDR^ mRNA (Figure 8d). Notably, embryos injected with the chimeric SF3B1^△IDR^‐FUS^IDR^ mRNA exhibited partly, rescue of developmental progression, and weaker than SF3B1^FL^ group (Figures 8d and S11). Taken together, we identify SF3B1 as an essential splicing factor that persistently localizes in nuclear puncta during mouse oocyte maturation and early embryogenesis. The condensation state of these puncta correlates with nuclear morphology and transcriptional activity [59], underscoring a critical role for SF3B1 LLPS‐mediated splicing regulation in mouse pre‐implantation development.

## 3 Discussion

Mammalian development begins with a totipotent zygote formed by fertilization of the sperm and oocyte. As cleavage proceeds, the zygote forms a blastocyst containing two molecularly and spatially distinct lineages that give rise to the embryo and extraembryonic tissues. Although these early cell fate decisions are well characterized, the post‐transcriptional regulatory mechanisms remain incompletely understood. Translational regulation is tightly associated with the splicing factor SRSF6, and dysregulation of splicing factors represents a major contributor to human oocyte aging [14]. Moreover, a splice site mutation in the meiotic gene REC114 can result in oocyte multinucleation and early embryonic arrest [60]. Therefore, elucidating RNA splicing mechanisms is crucial for defining mRNA isoform functions during early embryogenesis and for guiding strategies to address reproductive disorders arising from developmental failure.

Previous studies revealed that AS is very dynamic during mouse preimplantation development, with most changes in isoform usage occurring at the ZGA [10, 36, 61]. To investigate the role of AS in ZGA, PlaB, a natural antitumor analog of macrocyclic lactones that specially binds to the SF3B complex [62], was used to inhibit the function of splicing factors in zygotes, yet this treatment caused unexpected SF3B1 accumulation in 2‐cell embryo nuclei, and the effects were highly dependent on treatment timing. As a result, its regulatory impact on subsequent stages of embryonic development remains unexplored [32]. Therefore, our analysis of published RNA‐seq data revealed dynamic patterns of AS specifically from the 2‐cell to morula stages of mouse embryogenesis. We found blastomeres display the highest isoform diversity largely driven by ES, consistent with previous reports [10]. Previous studies have demonstrated that heterogeneity variety of specific ES splicing of CARM1 biases the first cell fate in mammalian early embryos [63]. Moreover, KD of factors involved in early steps of 5′or 3′ss recognition, such as components of U1 or U2 snRNP, more frequently affects ES events [64]. Recent reports further revealed that the spatial positioning of genes within distinct nuclear compartments is closely associated with their splicing patterns [65]. Therefore, we speculate that early embryonic cells require rapid remodeling of the transcriptome, and the relatively simple early steps of splicing confer an advantage, making ES an efficient regulatory strategy during developmental stage transitions. However, particularly during the 4‐ to 8‐cell and 8‐cell to morula transitions, the transcriptional dynamics of AS‐regulated genes were minimal. These findings suggest that, in addition to the dramatic changes in overall mRNA expression during cleavage, stably expressed transcripts are precisely regulated primarily through SE‐mediated splicing events. These observations indicate that mRNAs undergo temporally regulated alternative splicing, providing substantial functional diversification to genes and contributing to the regulatory mechanisms of embryonic development.

A screening study using an siRNA library to investigate the exit of mESCs toward the 2‐cell‐like state identified 49 top hits corresponding to five major complexes [19]. Notably, among these, the 23 core components of the spliceosome accounted for the largest proportion. These findings suggest that splicing‐mediated isoform diversity plays a critical role in the first cell fate decision, particularly during the later transitions of pluripotency. We performed cluster analysis of the RNA‐seq data and identified two genes, SF3B1 and LSM, that overlap between spliceosome factors upregulated after the 4‐cell stage and those 23 core spliceosomal components. We employed two approaches to investigate the function of splicing factors during early embryonic development: siRNA‐mediated knockdown of SF3B1 or SF3B6 in zygotes effectively reduced their mRNA levels, leading to embryonic arrest at the morula stage; PlaB treatment induced stage‐dependent developmental defects, causing 2‐cell arrest when applied at the 1‐ and 2‐cell stages, which disrupts of a large number of functional mRNA transcripts in ZGA progression consistent with previous research [36], but allowing partial blastocyst development exposure at the 4‐cell and morula stages. Previous studies have confirmed that AS operates independently of transcriptional regulation during development [33]. Our data revealed that SF3B1 knockdown in zygotes impaired de novo transcript production and caused transcriptome changes [64]. Thus, mouse development was shaped by an interplay of programs controlling gene expression levels and AS. Furthermore, siRNA‐mediated SF3B1 knockdown affected the extent of the embryonic cell cycle. These results indicate that proper SF3B‐mediated splicing is essential for early embryonic development.

Long before LLPS was formally defined, essential membrane‐less condensates had been recognized in the germline, where post‐transcriptional regulation is central to reproductive success [66], such as P granules [67], MARDO [68], and Ddx3xb [69]. Regulation of splicing factors is also mediated through LLPS [59]. In this study, we reveal the phase separation of SF3B1 during a critical period of early development. Specifically, we show that (1) SF3B1 possesses an intrinsic capacity for LLPS and forms nuclear condensates with tunable material properties through the coordinated action of its N‐terminal IDR and C‐terminal HEAT‐repeat domains. SF3B1 phase separation exhibits hallmark LLPS behaviors and is highly sensitive to key physicochemical parameters of the nuclear environment, including macromolecular crowding, ionic strength, protein concentration, and RNA abundance, indicating that its condensation state can dynamically respond to nuclear conditions; (2) during the NSN‐to‐SN transition in mouse oocytes, a profound chromatin configuration transition occurs. Concomitantly, SF3B1 redistributes from a diffuse speckled pattern in NSN oocytes to a more aggregated nuclear distribution in SN oocytes, suggesting that chromatin compaction, nuclear positioning [70] and transcriptional activity modulate the phase separation behavior of splicing factors [71]; (3) SF3B1 displays stage dependent reorganization, appearing as dispersed puncta in 1‐and 2‐cell embryos and becoming increasingly condensed from the 4‐cell stage to the blastocyst. Expression of the chimeric SF3B1^△IDR^‐FUS^IDR^ partially rescued the morula stage developmental arrest caused by SF3B1 knockdown. Together, SF3B1 undergoes LLPS and plays an essential role in mouse embryogenesis.

In summary, our findings provide pivotal insight into the role of SF3B1, a key alternative splicing factor, in controlling the first cell fate decision in the mouse embryo. We propose that SF3B1 undergoes LLPS in conjunction with developmental progression (Figure 8). Further studies will clarify how LLPS‐mediated regulation of alternative splicing contributes to embryonic development.

## 4 Methods

### 4.1 Mouse

All mice used in this study, including both C57BL/6 and DBA/2 strains, were sourced from Gempharmatech Co., Ltd. The colony was maintained under specific pathogen free (SPF) conditions with regulated environmental parameters: ambient temperature maintained at 20°C–22°C, relative humidity kept between 50% and 70%, and a 12 h light/12 h dark cycle. Standard chow and autoclaved water were available at all times. All experimental procedures were approved by the Animal Welfare Research Ethics Committee of Shanxi Medical University (Approval No.: SYDL2025038), with measures taken to minimize discomfort.

### 4.2 Cell Culture and Transfection

Human embryonic kidney (HEK293T) cells were cultured in DMEM(Gibco, L100) supplemented with 10% fetal bovine serum (FBS; BDBIO, F801) and 1% penicillin‐streptomycin (Solarbio, P1400) at 37°C in a humidified atmosphere containing 5 % CO_2_. For transfection, cells were seeded in 6‐well plates and grown to 70%–80% confluence prior to transient transfection with the plasmid using Lipofectamine 3000 Transfection Reagent (Thermo Fisher, L3000015) following the manufacturer's technical guidelines. Cells were collected 48 h post‐transfection for subsequent experimental analysis.

### 4.3 Oocyte and Embryo Collection and In Vitro Culture

For the collection of GV‐stage oocytes, 6 to 8 weeks old female C57BL/6 mice were injected with 10 IU PMSG (NSHF, 110254564). After 46–48 h, the mice were euthanized, and Germinal vesicle (GV) stage oocytes were released from antral follicles by manual tearing ovarian follicles in M2 medium (Nanjing AiBei Biotechnology, M1250) at 37°C. Oocytes were hand‐picked using a mouth‐controlled glass pipette and sequentially rinsed three times in pre‐warmed M2 medium, followed by two washes in fresh M16 medium (Sigma‐Aldrich, M7292). Oocytes were categorized as surrounded nucleolus (SN) or non‐surrounded nucleolus (NSN) according to germinal vesicle (GV) morphology. SN oocytes exhibited a centrally located GV surrounded by a distinct dark perinucleolar halo, whereas NSN oocytes displayed an eccentric GV with dispersed chromatin and absence of the halo. Only round, morphologically intact oocytes with a diameter ≥70 µm were collected within 30 s of retrieval. Subsequently, NSN or SN oocytes were transferred into 40 µL M16 microdroplets supplemented with 200 µM 3‐isobutyl‐1‐methylxanthine (IBMX; Sigma‐Aldrich, M8410), overlaid with light mineral oil (Sigma‐Aldrich, M8410).

For embryo collection, 6–8 weeks female C57BL/6 mice were injected intraperitoneally with 10 IU PMSG, followed 44–48 h later by 7.5 IU hCG (NSHF, 110251282), and then mated with DBA/2 males; copulation was verified by the detection of a vaginal plug. 14–16 h following hCG injection, early embryos were recovered from the ampullary of oviducts. Cumulus cells were removed by incubation in M2 medium containing 100 µg/mL hyaluronidase (Sigma, H1115000), and the embryos were subsequently transferred into 40 µL KSOM microdroplets (Nanjing AiBei Biotechnology, M1435) under mineral oil and cultured at 37°C in a humidified 5 % CO_2_ atmosphere. Embryos were harvested at defined intervals post‐hCG injection as follows: zygotes (20–22 h), early 2‐cell stage (30 h), late 2‐cell stage (46–48 h), 4‐cell stage (54–56 h), 8‐cell stage (68–70 h), morula (76–78 h), and blastocysts (96 h).

### 4.4 Microinjection

Recombinant mRNAs encoding GFP–SF3B1 constructs (full length, N‐terminal [residues 1–468], or C‐terminal [residues 469–1301]) were synthesized in vitro from linearized expression plasmids using the EasyCap T7 Co‐transcription Kit with CAG Trimer (Vazyme, DD4203) to generate 5′ capped transcripts, followed by polyadenylation using a Poly(A) Tailing Kit according to the manufacturer's instructions. SF3B1‐specific small interfering RNA (siSF3B1) and a negative control siRNA (siNC) were designed and synthesized by JTSBIO Co., Ltd. (Wuhan, China) (Table S2). The siRNAs were diluted to a final concentration of 20 µM in nuclease‐free water and stored at −80°C until use.

Microinjection was performed under an inverted microscope using the Eppendorf Inject Man 4 micromanipulation injection system. Embryos were placed in long microdrops of M2 medium covered with mineral oil. 5–10 pL of SF3B1 siRNA (20 µM) or recombinant mRNA (500 ng/uL) was slowly injected into the cytoplasm of each embryo and subsequently cultured in pre‐warmed KSOM medium. A negative control group was injected with an equal volume of nuclease‐free water to assess embryonic viability.

### 4.5 PlaB Treatment

Collected 4‐cell and morula stage embryos were transferred to KSOM medium containing 50 nM PlaB (GLPBIO, GC18780) or DMSO (YEASEN, 60313ES60) as a vehicle control for in vitro treatment. Embryo development was monitored to assess progression from 4‐cell to 8‐cell stage, and from morula to blastocyst. Following the treatment, morphologically normal embryos from each group were collected for RNAseq.

### 4.6 Plasmid Construction

To assemble the full‐length mouse SF3B1 cDNA, the CDS was first retrieved from NCBI (RefSeq: NM_011365.4). Initial cloning attempts failed because the SF3B1 wild‐type sequence exerted strong toxicity in bacteria. We therefore codon‐optimized the entire open reading frame for E. coli expression and commercially synthesized the gene (Table S2).

Three pEGFP‐C1‐derived constructs, FL‐SF3B1, SF3B1(1‐468) and SF3B1(469‐1301), were generated by Gibson assembly. The vector was digested with Bgl II/Hind III (Beyotime), gel purified (TIANGEN, DP209) and combined with PCR fragments using DNA Assembly Mix Ultra (Yugong Biology) according to the manufacturer's protocol. All reactions were transformed into competent EPI400 cells; positive clones were verified by Sanger sequencing. All primers used for cloning are provided in (Table S2).

### 4.7 Immunofluorescence Staining

Mouse embryos at various developmental stages were collected and fixed with 4% paraformaldehyde (Beyotime, P0099) at room temperature for 15–30 min. After 20 min of permeabilization in 0.4% Triton X‐100/PBS, embryos were blocked in blocking buffer (2% BSA, 0.1% Tween, and 0.01% Triton X‐100 in PBS) for 1 h at room temperature. Embryos were then incubated overnight at 4°C with rabbit anti‐SF3B1 primary antibody (Abcam, ab204916, 1:500), washed three times with 0.1% Tween 20 + 0.01% Triton X‐100 in PBS, and labeled for 2 h at room temperature with Alexa Fluor 488‐conjugated donkey anti‐rabbit IgG (Abcam, ab150061, 1:500) in the dark. Following final washes, samples were mounted in Antifade Mounting Medium with DAPI (Beyotime, P0131), imaged on a Zeiss LSM900 confocal microscope, and fluorescence signal intensity quantified with ImageJ software.

### 4.8 5‐Ethynyl Uridine (EU) Incorporation Assay

To monitor nascent RNA synthesis, embryos at various developmental stages were cultured in KSOM medium containing 1 mM EU for 2 h at 37°C under 5% CO_2_ atmosphere. Following metabolic incorporation, embryos were fixed in 4% paraformaldehyde for 30 min at room temperature and subsequently permeabilized with permeabilization buffer (0.3% Triton X‐100 in PBS) for 20 min. The incorporated EU was fluorescently labeled using the BeyoClick EU RNA Synthesis Kit with AF488 (Beyotime, R0301S) according to the manufacturer's instructions, with specimens incubated for 30 min at room temperature in darkness. After extensive washing, embryos were mounted using Antifade Mounting Medium with DAPI (Beyotime, P0131). Confocal imaging was performed using a Zeiss LSM900 microscope with uniform exposure settings applied across all samples to facilitate comparative analysis of transcriptional activity.

### 4.9 QRT‐PCR

To quantitatively determine the mRNA expression levels of target genes in mouse embryos at different developmental stages and the corresponding SF3B1‐knockdown (SF3B1‐KD) embryos, real‐time quantitative polymerase chain reaction (RT‐qPCR) was employed. For each sample, 10 wild‐type mouse embryos (covering zygote, 2‐cell, 4‐cell, and 8‐cell stages) and the corresponding SF3B1‐KD embryos were collected for cDNA synthesis. RT‐qPCR reactions were performed using PerfectStart Green qPCR SuperMix (+Universal Passive Reference Dye) (TRANS, Cat. No.: AQ602) on a Thermo Fisher Scientific QuantStudio 3 Real‐Time PCR System. To account for variation in input template quantity, H2afz served as the endogenous reference gene for normalization. All primer sequences are provided in the Table S2. Every sample was run in triplicate, and the assay was replicated at least 3 independent experiments. Relative mRNA abundance was quantified by 2^(–ΔΔCt) and expressed as mean ± SEM.

### 4.10 Western Blot

HEK293 cells transfected with pEGFP‐C1‐SF3B1 plasmid were harvested 24 h post‐transfection. Cells were lysed using enhanced RIPA lysis buffer (Beyotime, P0013B) and denatured at 95°C for 10 min. Proteins were separated by SDS‐PAGE and transferred onto polyvinylidene difluoride membranes (Merck Millipore, IPVH00010). Membranes were blocked with TBST buffer supplemented with 5% non‐fat milk (BBI,A600669) for 2 h at room temperature, followed by overnight incubation at 4°C with primary antibodies: mouse monoclonal GFP antibody (Beyotime, AG281, 1:2000), rabbit polyclonal SF3B1 antibody (ABclonal, A15801, 1:1000), and GAPDH as a loading control (YEASEN, 30202ES60, 1:5000). Membranes were three washes with TBST and incubated for 1 h at room temperature with Goat Anti‐Rabbit IgG H&L HRP‐conjugated (Bioss, BC10268988, 1:10,000) and Goat Anti‐Mouse IgG H&L/HRP (Bioss, BC12124606, 1:10,000) secondary antibodies. Immunoreactive bands were visualized using ECL chemiluminescence detection reagent (Meilunbio, MA0186), and densitometric quantification was performed using ImageJ software.

### 4.11 Total RNA‑Seq Library Construction

RNA‐seq libraries were generated from embryonic samples using the Smart‐seq2 protocol (Picelli et al., Nat. Protoc. 2013). For each group (siNC and siSF3B1), 10 embryos at the 4‐cell and morula stages were collected, with three biological replicates per stage. Embryos were washed three times with phosphate‐buffered saline (PBS) containing 0.2% (w/v) bovine serum albumin (BSA) to remove debris and residual medium, then transferred into 200‐µL RNase/DNase‐free PCR tubes containing 0.1% Triton X‐100, 10 mM dNTPs, 10 µM oligo‐dT primer, and RNase inhibitor (RiboLock RNase, Thermo Fisher Scientific, EO0382). Samples were lysed at 72°C for 5 min. First‐strand cDNA synthesis, template switching, Tn5 tagmentation, and library construction were performed according to standard Smart‐seq2 procedures. Libraries were sequenced on an Illumina HiSeq 6000 platform.

### 4.12 Transcriptome and Splicing Events Analyses

RNA sequencing Raw data were trimmed into 50 bp reads and mapped to the mouse reference genome (mm10) using HISAT2 (v2.1.0) with default parameters. Uniquely mapped reads were assembled into transcripts using reference annotations and Cufflinks (v2.2.1). Relative gene expression levels were quantified using FPKM, Genes with a multiple of |log2fold change| > 2 and *p* value < 0.05 were considered as differentially expressed genes. ASEs were identified using replicate multivariate analysis of transcript splicing (rMATS), which detected five major types of ASEs. Events with a false discovery rate (FDR) below 0.05 were considered significant.

### 4.13 LACE‐seq Library Generation, Sequencing

The experiment was performed following the LACE‐seq protocol with minor modifications. In brief, approximately 300 embryos at the 4‐cell and morula embryos were manually picked under a stereomicroscope using fine glass needles. The zona pellucida was removed, and the embryos were washed twice with BSA/PBS solution (0.1% BSA in 1× PBS) to eliminate debris. The cleaned embryos were then resuspended in PBS in new RNase/DNase‐free EP tubes. The cells were irradiated twice with UV‐C light (400 mJ/cm^2^) on ice to crosslink RNA–protein complexes, then lysed in washing buffer supplemented with RNase inhibitor (RiboLock RNase, Thermo Fisher Scientific, EO0382) and RQ1 DNase (Promega, M6101). The SF3B1–RNA complexes were subsequently immunoprecipitated using SF3B1 antibody (Abcam, ab172634) conjugated to magnetic beads for enrichment, with normal IgG used as a negative control. The immunoprecipitated RNA–protein complexes were lightly fragmented on beads using micrococcal nuclease (MNase), followed by 3′ RNA dephosphorylation with FastAP alkaline phosphatase (Thermo Scientific, EF0651). Subsequently, 3′ linker ligation was performed using T4 RNA ligase 2, truncated (NEB, M0242) and a pre‐adenylated linker (/5rApp/NNNNAGATCGGAAGAGCGTCGTGTAGGGAAAGAGTGT/3ddC/). Reverse transcription was carried out with SuperScript II Reverse Transcriptase (Thermo Fisher Scientific) using the primer 5′‐Bio‐GATCACTAATACGACTCACTATAGGGACACTCTTTCCCTACACGACGCTCTTCCGATCT‐3′. RNA strands in RNA/DNA hybrids were digested with RNase H (Thermo, EN0201), and the remaining cDNA was captured using C1 beads. A second linker (p‐NNNNNNNNCGATTGNNNNAGATCGGAAGAGCACACGTCTGAA‐NH_2_) was ligated to the cDNA using T4 RNA ligase, replacing the poly(A) tailing step used in the original LACE‐seq protocol. The resulting single‐stranded DNA (ssDNA) was pre‐amplified and purified using AMPure XP beads (Beckman Coulter, A63882). The purified DNA served as a template for in vitro transcription using T7 RNA polymerase (Thermo Fisher, EP0113), followed by RNA purification with Agencourt RNA Clean beads. The purified RNA was then reverse‐transcribed into cDNA, and PCR amplification was performed to generate sequencing libraries. The final LACE‐seq libraries were sequenced on an Illumina HiSeq 2500 platform according to the manufacturer's instructions. We used at least 300 embryos for the high quality data of SF3B1 LACE‐seq. Each condition was analyzed with two independent biological replicates.

### 4.14 LACE‐seq Data Processing, Alignment, and Peak Calling

Adapter trimming and quality filtering were performed using Cutadapt (v5.1) in paired‐end mode. Reads were trimmed for adapter sequences, quality‐filtered with a minimum Phred score of 20, and reads shorter than 18 bp were discarded. Reads containing >50% ambiguous nucleotides (N) were excluded. Poly‐G sequences (≥12 consecutive guanines) were removed in a second trimming step.

Cleaned reads were aligned to the Mus musculus reference genome (GRCm38.102, Ensembl) using Bowtie2 (v2.5.4) in paired‐end local alignment mode. Peak calling was performed using Piranha (v1.2.1) with the following parameters: ‐s ‐b 50 ‐i 50 ‐d Zero Truncated Negative Binomial Regression ‐p 0.05. The resulting peaks were processed using HOMER (v5.1), where annotation was performed with annotatePeaks.pl and motif enrichment was analyzed using findMotifsGenome.pl with the ‐size 25 option.

### 4.15 Protein Expression and Purification

The SF3B1‐IDR fragment was cloned into a pET vector with an N‐terminal GST tag and C‐terminal His tag. Escherichia coli BL21 (DE3) cells were transformed with the recombinant plasmid. A single colony was cultured in LB medium (with appropriate antibiotics) at 37°C with shaking until OD600 reached 0.6–0.8. Protein expression was induced by adding IPTG to a final concentration of 0.3 mM, followed by overnight incubation at 37°C. Bacterial cells were harvested by centrifugation (4000 rpm, 20 min) and resuspended in lysis buffer (50 mM Tris‐HCl pH 7.5, 500 mM NaCl, 5 mM imidazole,1 mM PMSF). Cells were disrupted using a high‐pressure homogenizer, and lysates were centrifuged (18,000×g, 40 min, 4°C). The supernatant was loaded onto a Ni‐NTA HiTrap affinity column. The column was washed extensively with wash buffer (50 mM Tris‐HCl pH 7.5, 500 mM NaCl, 5 mM imidazole) to remove contaminants. Target proteins were eluted with elution buffer (50 mM Tris‐HCl pH 7.0, 500 mM NaCl, 5% glycerol, 500 mM imidazole), and fractions were analyzed by SDS‐PAGE for purity and concentration. Purified proteins were dialyzed into storage buffer (50 mM Tris‐HCl pH 7.5, 500 mM NaCl), aliquoted, and stored at −80°C.

### 4.16 Protein Labelling With a Fluorescent Dyes

Purified proteins were exchanged for fluorescent labeling into 100 mM NaHCO_3_ pH 8.3, using a HiTrap desalting column and concentrated to 10 mg/mL before the reaction. The Alexa488‐ester was dissolved in DMSO and incubated with the corresponding protein at room temperature for 1 h. The Alexa Fluor 488 to protein molar ratio was 1:1. The reaction was quenched by 200 mM Tris‐HCl pH 8.3, and the labeled proteins were further changed into the buffer (50 mM Tris‐HCl pH 7.5 and 100 mM NaCl) by the HiTrap desalting column.

### 4.17 In Vitro Phase Separation Assay

Purified proteins were diluted to specified concentrations in 50 mM Tris–HCl buffer (pH 7.5) with defined salt concentrations. LLPS was induced by adding predetermined concentrations of PEG8000 or total RNA. Samples were mixed in low‐binding tubes and incubated on ice for 5 min to initiate phase separation. For imaging, 5 µl of each protein solution was mounted on a glass slide, covered with a coverslip, and visualized using a Zeiss LSM 900 confocal microscope with a 63× oil immersion objective (GFP excitation: 488 nm; emission: 509 nm). For RNA‐induced LLPS, total RNA was isolated from HEK293T cells using TRIzol reagent (Thermo Fisher Scientific) following the manufacturer's protocol. RNA concentration was quantified via a Nanodrop 2000 spectrophotometer (Thermo Fisher Scientific) by absorbance at 260 nm, and purity was verified by A260/A280 ratio (target: 1.8–2.0).

### 4.18 Confocal Imaging

HEK293T cells were cultured in glass‐bottom dishes (Nest) and transfected with the indicated plasmids for confocal imaging. Plasmids were transfected into cells at 50%–60% confluence using Lipofectamine 3000 Transfection Reagent (Thermo Fisher, L3000015) following the supplier's instructions. At 24 h post‐transfection, cells were imaged using a Zeiss LSM 900 confocal microscope equipped with a 63× oil‐immersion objective. Images and data were acquired and processed using Zen Black software (Zeiss).

For the fluorescence recovery after photobleaching (FRAP) assay, HEK293T cells were cultured in glass‐bottom dishes and transfected with the indicated plasmids as described above. GFP fluorescence signals in predefined regions of interest (ROIs) were bleached using a 488 nm laser. Post‐bleaching images were acquired at 1‐s intervals. Relative fluorescence intensity was calculated with the pre‐bleaching fluorescence intensity set as the reference. The fluorescence intensity difference between the pre‐bleaching stage and time 0 (immediately after the bleaching pulse) was normalized to 100%.

## Author Contributions

K.Z. designed the study and wrote the manuscript. T.H. provided bioinformatics support. Y.C. and Y.Z. collected embryonic samples and performed oocyte collection, embryo culture, imaging, phase‐separation assays, RNA‐seq, and LACE‐seq. S.Z., W.H., and J.Z. conducted gene editing and downstream bioinformatics analyses. P.L. and Y.Z. provided critical reagents and contributed to manuscript revision. K.Z., Z.L., Q.S., and J.X. secured funding, coordinated the project, and approved the final version. All authors read and approved the manuscript.

## Funding

This work was supported by the National Natural Science Foundation of China (32370641 to K.Z.; 32300580 to L.Y.C.); National Natural Science Foundation of China Regional Innovation and Development Joint Fund (Grant No. U23A20420 to J.X.).

## Conflicts of Interest

The authors declare no conflict of interest.

## Supporting information




**Supporting File 1**: advs77605‐sup‐0001‐SuppMat.docx.

**Supporting File 2**: advs77605‐sup‐0002‐VideoS1.avi.

**Supporting File 3**: advs77605‐sup‐0003‐TableS1.csv.

**Supporting File 4**: advs77605‐sup‐0004‐TableS2.xlsx.

**Supporting File 5**: advs77605‐sup‐0005‐TableS3.xlsx.

## Data Availability

Publicly available datasets used in this study were retrieved from the NCBI BioProject database (PRJNA1395506 for LACE‐seq data and PRJNA1391903 for RNA‐seq data). Source data are provided with this paper, and experimental data are included in the Supplementary Tables.
