# Supplementary material for: Phase Separation of SF3B1 Serves as a Critical Post‐Transcriptional Regulator During Early Mouse Embryogenesis
Source: Adv Sci (Weinh). 2026 Sep 8:e77605. Online ahead of print. doi: 10.1002/advs.77605 (PMC13554399; doi:10.1002/advs.77605)
Supplement: Supplementary file 1 [file ADVS-9999-e77605-s003.docx]

Supplementary Fig.1. Dynamic landscape of alternative splicing during mouse pre-implantation development.

**a** Heatmap showing the distribution of five alternative-splicing event types detected between consecutive stages: 2-cell to 4-cell, 4-cell to 8-cell, and 8-cell to morula transitions. Data derived from total RNA-seq; event classes (MXE, SE, A3SS, A5SS, RI) were called with rMATS (false-discovery rate (FDR) < 0.05, |ΔPSI| > 0.1). Numbers above bars indicate total events per transition. **b** Scatter plots of gene-level changes across 2- to 4-cell transitions. Gray dots indicate non-significant genes; red dots mark up-regulated genes, blue dots mark down-regulated genes. The y-axis displays log₂(baseMean) expression level. Significance criteria: FDR < 0.05 and absolute |(log₂FC)| ≥ 2 (FC, fold change). **c** Stacked-bar plot of differential alternative-splicing events (ASEs) of up, down, and no changed DGE across pre-implantation transitions: 2C–4C, 4C–8C and 8C–morula. Colors denote ASE different class; bar height represents log₂-transformed event counts. Up, inclusion increase; Down, inclusion decrease (FDR < 0.05, |log₂FC| ≥ 2), denoting significant gene-expression changes. **d** Major PSI (percent-spliced-in) trajectory clusters identified by Mfuzz clustering of alternative splicing events across mouse 2-cell, 4-cell, 8-cell, and Morula stages; Lines show median PSI ± SEM for each of 11 clusters; n = number of splicing events within each cluster. Distinct temporal patterns reveal stage-specific splicing regulation. **e** The pie chart illustrates the classification of 4,199 alternative splicing events based on their PSI dynamics: PEAK (peak-like AS pattern; 31.6 %), SHIFT (shift-like AS pattern; 41.7 %), and Others (26.7 %).

Supplementary Fig.2. Functional impact and enrichment analysis of alternative splicing events.

**a-c** Stacked bar plots illustrating the relative proportions of predicted functional categories across five different ASEs across early embryonic developmental transitions: 2-cell to 4-cell(**a**), 4-cell to 8-cell(**b**), and 8-cell to morula (**c**) using SpliceImpactR. Color legend: Match (no change in protein-coding potential), protein_coding (no frameshift), FrameShift (frameshift-inducing), Rescue (rescued frameshift), onePC (one premature termination codon), and noPC (no premature termination codon). **d-f** GO analysis on splicing events annotated as FrameShift, onePC and noPC for the corresponding developmental transitions shown in a-c (d for a, e for b, f for c).

Supplementary Fig.3. Expression landscape of splicing factors dynamics across early embryogenesis.

**a** Heatmap of candidate splicing factors expression retrieved from UniProt (https://www.uniprot.org/) across ten developmental stages (FGO, MI, MII, PN3, PN5, E2C, L2C, 4C, 8C, ICM). Expression values were z-score row-normalized and clustered by Euclidean distance with complete-linkage. The gene dendrogram was cut into five clusters (cutree k = 5). **b** Line plots showing the mRNA profiles of SF3b complex factor in various developmental stages of mouse preimplantation embryos were examined by RNA-seq. **c-d** Bubble plot showing top enriched Gene Ontology biological process (BP) terms for Cluster 4(**c**) and Cluster5(**d**) genes. X‑axis shows enriched gene count; color denotes −log₁₀(adjusted P‑value).

Supplementary Fig.4. phenotypes of AS loss of function in early mouse embryos.

**a-b** Schematic of the experimental pipeline for generating SF3B1-knockdown(KD) and PlaB-treated embryos. Zygotes were microinjected with siRNA against SF3B1 or non-targeting control, cultured to 4-cell or Morula stage, and collected for RNA-seq(**a**). In parallel, embryos were exposed to 50 nM PlaB or DMSO from the zygote, 2-cell, 4cell, and morula stages onward(**b**). **c** Immunofluorescent staining images for SF3B1 in 2-cell embryos after siNC or siSF3B1 microinjection (Five independent experiments). Scale bar: 20μm. Relative SF3B1 intensity was quantified with ImageJ and is expressed as mean ± SEM; groups were compared by two-tailed unpaired Student’s t-test. **d-g** Representative images of mouse embryonic development. Embryos at the 1-cell (**d**), 2-cell (**e**), 4-cell (**g**), and morula (**f**) stages were treated with PlaB to block SF3B1 function; DMSO served as the vehicle control. Scale bar, 100 μm. **h** Representative images of embryonic development after microinjection of mCherry mRNA plus SF3B1-targeting siRNA into one blastomere of 2-cell embryos. Red fluorescence marks the progeny of the injected cell. Scale bar, 100 μm.

Supplementary Fig.5. Transcriptome reliability and differential landscape upon SF3B1 loss or PlaB treatment during pre-implantation development.

**a-c** Heatmaps of Spearman correlations among total-transcriptome profiles for 4-cell and Morula embryos under SF3B1-KD vs control (a), and after PlaB treatment vs DMSO at 4-cell (**b**) and Morula (**c**) stages. **d** Scatter plots show pairwise concordance of RNA-seq biological replicates for 4-cell and Morula embryos under control, SF3B1-KD and PlaB conditions. **e** Scatter plot of relative transcript levels in 4-cell embryos after PlaB treatment (total RNA-seq). Blue, down-regulated genes; pale red, up-regulated genes. **f** Scatter plot illustrating the correlation of gene expression levels, quantified as log₂‑transformed base mean values, between SF3B1‑KD and PlaB‑treated mouse morula embryos. Individual genes are shown as blue dots, the red line denotes the linear regression fit, and the Pearson correlation coefficient was calculated as R = 0.869. **g** GO enrichment analysis of differentially expressed genes (DEGs) in SF3B1-KD at 4-cell embryos.

Supplementary Fig.6. SF3B1 depletion perturb totipotent‑pluripotent factors without affecting other spliceosome component.
**a-b** Scatter plot of RNA-seq gene-expression changes in Morula embryos upon SF3B1-KD (log₂FC ≥ |2|, FDR < 0.05). Gray dots indicate non-significant genes; red dots mark up-regulated genes; blue dots mark down-regulated genes. Yellow dots highlight pluripotent(a) and totipotent genes(b). **c** Heatmap showing the selected totipotency and pluripotency genes of RNA-seq derived relative expression (log₂-normalized) altered in PlaB-treated morula. Color intensity represents relative expression levels, with blue indicating lower expression and red indicating higher expression. **d-f** Scatter plots of transcriptomic alterations in SF3B1-KD 4-cell embryos(d), SF3B1-KD morula(e), and PlaB-treated morula(f). Yellow dots highlight SF3B family genes. Significance criteria: FDR < 0.01 and |(log₂FC)| ≥ 1.

Supplementary Fig.7. SF3B1 loss disrupts blastocyst-lineage programs by rewiring transcription factors, destabilizing tight-junction components, and enhancing Wnt signal and stem cell differentiation.
**a-b** Scatter plots show transcription factors (TFs) expression change at the 4-cell (a) and Morula (b) stages after SF3B1 depletion. Significance thresholds: |log₂FC| > 1 and FDR < 0.05. Red dots, SF3B1-KD/NC group; blue dots, PlaB/DMSO treated group; Circle size scales with −log₁₀(FDR); larger symbols denote higher significance. **c-e** Scatter plots of significantly altered genes (FDR < 0.05, |log₂FC| > 1) encoding tight-junction components (c), Wnt-pathway members and stem-cell differentiation regulators (d) in morula stages following SF3B1 depletion. Y-axis: baseMean expression level (normalized counts); X-axis: log₂FC. **f-g** Representative brightfield images show preimplantation development to blastocyst stage. SF3B1-KD(f) and PlaB-treated morula(g) were cultured with or without the Wnt inhibitors IWR-1 or IWP-2. Scale bar: 100 μm. **h** Immunofluorescent staining images for TUNEL (green) and DAPI (blue) in morula embryos after siNC or siSF3B1 microinjection. Scale bar: 20μm. **i-j** Immunofluorescent staining images for γH2AX(i), α-Tubulin (j) and DAPI (blue) in PlaB-treated embryos at 86 h post-hCG. Scale bar: 20μm. **k** Immunofluorescent staining images for Pol2(ser2) (green) and DAPI (blue) in morula embryos after siNC or siSF3B1 microinjection. Scale bar: 20μm. **l** RT-qPCR analysis of Centromeric satellite RNAs MinSAT (centromeric) and MajSAT (pericentromeric) in WT and SF3B1-KD morula embryos. Transcript levels were normalized to endogenous GAPDH and presented as mean ± SD of three biological replicates. P values: 6.00×10⁻⁵ (MinSAT), 4.99×10⁻⁴ (MajSAT; Student’s t-test).

Supplementary Fig.8. Identify of SF3B1 impact on alternative splicing.

**a-b** Heatmap of normalized expression levels for transcripts undergoing MXE, SE, or A3SS alternative splicing events in the Morula stage(a) and 4-cell stages(b). **c** Workflow for identifying SA and SR events. **d** Bar plot showing the number of DEGs classified as upregulated(green), downregulated (red), or unchanged(gray) in SA and SR. **e** Venn diagram depicting the overlap between alternative splicing events (ASEs) and DEGs in siSF3B1 vs. siNC at the 4-cell stage (top). GO enrichment analysis of the 115 overlapping genes is shown below.

Supplementary Fig.9. Validation of abnormal splicing and LACE-seq-derived intronic binding of SF3B1 target genes

**a** Sashimi plots depict read coverage and exon–exon junction usage for *Mdm2*. **b** Semiquantitative RT-PCR (*n* = 3 per group) mapped *Mdm2* ectopic splicing in SF3B1-KD morula and representative gels combined with pooled quantification of the specified fragment are showed accordingly. **c-f** As in a and b, but for Ezh2: Sashimi plots (c, e) and semiquantitative RT-PCR validation (d, f) with quantification. **g** Representative image represents embryonic development from the 2-cell stage to the blastocyst stage. Mouse embryos at zygotic stage were microinjected with Ccnb1-mCherry or truncated Ccnb1^△C^-mCherry mRNA. Scale bar: 100 μm. **h** Fluorescent images for Ccnb1-mCherry and Ccnb1^△C^-mCherry (Red) and DAPI (blue) in morula embryos. Scale bar: 20μm. **i** RT-qPCR validation of *Elf2* intronic RNA enrichment in SF3B1 LACE-seq samples. Fold enrichments were calculated relative to the input samples and normalized to the IgG control. Data are presented as the mean ± SD (n = 2 biologically independent replicates).

Supplementary Fig.10. LLPS property and subnuclear localization of SF3B1 via its IDR

**A** Domain structure and intrinsic disorder tendency of SF3B1. The top panel shows the domains of SF3B1, along with catGRANULE analysis. **b** Coomassie-stained SDS-PAGE analysis of recombinant SF3B1^IDR^ protein after Ni-NTA affinity purification. The arrow marks the highest-purity elution fraction (red box), which was selected for subsequent concentration and liquid-liquid phase separation experiments. Molecular weight standards (kDa) are indicated on the left. **c** Microcentrifuge tubes showing solutions. The “Control” contains T50N100 buffer (50 mM Tris-HCl, 100 mM NaCl, pH 7.5) supplemented with 10% PEG8000. The “SF3B1^IDR^” sample contains 10 μM SF3B1^IDR^ in the same buffer, which exhibits visible turbidity. **d-f** Fluorescence imaging of 293T cells co‑expressing GFP-SF3B1 and mCherry‑PSPC1(d), PML(e), or SRSF2(f) with DAPI nuclear staining (blue). Line‑scan intensity analysis (ImageJ Fiji) along the dashed lines generates corresponding intensity‑distance profiles. Scale bar: 5 μm.

Supplementary Fig.11. Developmental rate statistics for SF3B1 rescue constructs in siSF3B1 embryos

**a** Statistical evaluation of siSF3B1 injection combined with GFP-SF3B1^FL^, GFP-SF3B1^IDR^or GFP-SF3B1^ΔIDR-^FUS^IDR^ on embryonic developmental rate. n = 3 biologically independent replicates. Data are mean ± SD. P‑values were determined by two‑tailed Mann–Whitney U test.
